# Dietary manganese, type 2 diabetes, and cardiovascular disease: A UK Biobank cohort study and meta-analysis of over 270,000 individuals

**DOI:** 10.1016/j.jnha.2025.100754

**Published:** 2025-12-10

**Authors:** Gebretsadkan Gebremedhin Gebretsadik, Bo Yang, Andrea J. Glenn, Ai-Min Yang, Jie Li, Vicky Wai-Ki Chan, Man-Sau Wong, Simin Liu, Ka-Hei Kenneth Lo

**Affiliations:** a Department of Food Science and Nutrition, The Hong Kong Polytechnic University, Hong Kong Special Administrative Region, China; b Department of Nutrition and Dietetics, School of Public Health, Mekelle University, Mekelle, 1871, Ethiopia; c Department of Epidemiology and Biostatistics and the Center for Global Cardiometabolic Health & Nutrition (CGCHN), Joe C. Wen School of Population and Public Health, Susan & Henry Samueli College of Health Science, University of California, Irvine, California 92697, USA; d Center for Global Cardiometabolic Health, Department of Epidemiology, School of Public Health, Brown University, Providence, Rhode Island, 02912, USA; e Department of Nutrition and Food Studies, New York University, New York, 10012, USA; f Department of Nutrition, Harvard T.H. Chan School of Public Health, Boston, MA, 02138, USA; g Department of Medicine and Therapeutics, The Chinese University of Hong Kong, Prince of Wales Hospital, Hong Kong Special Administrative Region, China; h Hong Kong Institute of Diabetes and Obesity, The Chinese University of Hong Kong, Prince of Wales Hospital, Hong Kong Special Administrative Region, China; i Global Health Research Centre, Guangdong Provincial People’s Hospital, Guangdong Academy of Medical Sciences, Southern Medical University, Guangzhou, 510515, China; j School of Public Health, Southern Medical University, Guangzhou, 510515, China; k Research Centre for Chinese Medicine Innovation, The Hong Kong Polytechnic University, Kowloon, Hong Kong Special Administrative Region, China; l Research Institute for Smart Ageing, The Hong Kong Polytechnic University, Hong Kong Special Administrative Region, China; m Department of Epidemiology and Biostatistics and the Center for Global Cardiometabolic Health & Nutrition (CGCHN), Joe C. Wen School of Population and Public Health; The Mary & Steve Wen Cardiovascular Division, Department of Medicine, Susan & Henry Samueli College of Health Science, University of California, Irvine, California 92697, USA

**Keywords:** Cardiovascular disease, Cohort study, Dietary intake, Manganese, Meta-analysis, Type 2 diabetes

## Abstract

**Objectives:**

To examine the association of dietary Manganese (Mn) intake with type 2 diabetes (T2D) incidence, total cardiovascular disease (CVD), and CVD mortality by analyzing data from the UK Biobank and conducting a meta-analysis of available prospective cohorts.

**Design:**

Prospective analysis of a primary cohort with a dose-response meta-analysis of prospective cohorts.

**Setting:**

The UK Biobank cohort and the meta-analysis of prospective cohorts.

**Participants:**

UK Biobank participants aged 40–69 years at baseline were enrolled between 2006 and 2010 and followed until December 2022. We included 165,194 participants in T2D analytic cohort and 164,111 individuals in CVD analytic cohort. Our systematic review and meta-analysis of six studies comprised over 270,000 participants.

**Exposure:**

Dietary manganese (Mn) intake.

**Measurements:**

The outcome measurements were T2D incidence, total CVD, and CVD mortality. Dietary intake was assessed using 24-h dietary instrument. Cox proportional hazards models were used to assess associations of Mn intake with T2D and CVD risk. Effect estimates were presented in hazard ratios (HR) with 95% confidence intervals (CI). In meta-analysis, a pooled risk for a 1 mg/day increase in Mn intake was estimated using restricted maximum likelihood (REML).

**Results:**

High Mn intake (Q5) was not significantly associated with lower risk of T2D as compared to Q1 (adjusted HR 0·91; 95% CI 0·82, 1.01, P_trend_ = 0·07). The dose-response meta-analysis revealed a 4% reduction in T2D risk with each mg/day increase in Mn intake (pooled RR 0·96; 95% CI 0·94, 0·99), with potential non-linearity (P_nonlinear_< 0.01). Q5 Mn intake was not significantly associated with reduced risk of CVD (adjusted HR 0·99; 95% CI 0·92, 1.05; *P_trend_* = 0·61) or CVD mortality (adjusted HR 0·85; 95% CI 0·64, 1.13; *P_trend_* = 0·66).

**Conclusions:**

Our meta-analysis suggested that increasing Mn intake may lower T2D risk, potentially exhibiting a dose-response non-linear pattern, although not corroborated by UK Biobank analysis.

## 1 Introduction

Type 2 diabetes (T2D) has emerged as a global health crisis, affecting 828 million adults worldwide in 2022 [1]. In 2021, the International Diabetes Federation reported that 537 million adults were affected by diabetes, with forecasts suggesting this figure could reach 783 million by 2045 [2]. Moreover, cardiovascular diseases (CVD) have emerged as a primary cause of mortality and morbidity globally, with 523 million cases and 18.6 million fatalities reported in 2019 [3]. Nutrition plays critical role in preventing T2D and CVD, which are interconnected conditions sharing common risk factors such as obesity, unhealthy eating patterns, lack of exercise, and alcohol intake [4].

Manganese (Mn) is an essential element that plays an indispensable role in the normal metabolic processes of amino acids, lipids, proteins, and carbohydrates [5]. Mn is chiefly derived from sources such as nuts, grains, fruits, leafy green vegetables, and caffeinated beverages [6,7]. It acts as a cofactor for several enzymes such as arginase, glutamine synthetase, pyruvate carboxylase, and Mn superoxide dismutase (Mn-SOD) [8]. In the United States, for individuals aged ≥19 years, the adequate intake of Mn was 1·8 mg/day for women to 2·3 mg/day for men [6]. Although not being a common condition, deficiency of Mn has been related to increased risk of mitochondrial oxidative stress, impaired glucose and lipid metabolism, and metabolic syndrome [9].

In view of its potential cardiometabolic benefits, several cohorts have studied the links between dietary Mn intake and the risk of T2D or CVD. A prospective cohort study of postmenopausal women in the United States revealed a 30% lower risk of T2D comparing those with the highest quintile of Mn intake to the lowest [10]. Besides, a study of two prospective cohorts from China found inverse associations between dietary Mn and T2D risk and HbA1C levels, and these associations were mediated by high plasma levels of Mn, increased Mn-SOD, and decreased 8-hydroxydeoxyguanosine [11]. Moreover, a study of Japanese adults reported a strong inverse association between dietary Mn and T2D risk in women but not in men [12], indicating a potential sex-specific association. As for CVD risk, a Japanese cohort study found a significant association between higher Mn intake and reduced risk of CVD mortality [13]. In contrast, another cohort from Iran observed no significant association between Mn intake and CVD mortality, when comparing individuals in the highest and lowest quintiles of intake (HR 1·07; 95% CI: 0·87, 1·20) [14].

Previous literature has shown inconsistent results regarding the association between dietary Mn and the risk of T2D and CVD, highlighting the need for a comprehensive review to summarize existing evidence. To fill this gap, we conducted an analysis of the United Kingdom Biobank (UK Biobank) data, supplemented by a meta-analysis of available prospective cohorts.

## 2 Material and methods

### 2.1 UK Biobank

#### 2.1.1 Study design and participants

This study drew on data from the UK Biobank, a large-scale prospective cohort study that recruited >500,000 individuals aged 40–69 years from various regions of the United Kingdom between 2006 and 2010. Participants completed detailed questionnaires on diet, lifestyle factors, and medical history at baseline.

From the total UK Biobank population of 502,370, we excluded 291,420 participants due to the absence of dietary records. Among the remaining 210,950 participants with at least one dietary record, 38,082 were further excluded due to missing data on race/ethnicity, education status, Townsend deprivation index, physical activity, smoking, alcohol drinking, BMI, energy intake, baseline medication, and family history of T2D/CVD, and due to implausible total energy intake (≤600 kcal/day or ≥5000 kcal/day). We have analyzed the differences in several characteristics between included and excluded participants with dietary intake data (Supplementary Table S1).

For the T2D analytic cohort, 6,971 participants with T2D at baseline and 703 participants diagnosed with T2D before dietary assessment were excluded, yielding a final analytic cohort of 165,194 participants. For the CVD analytic cohort, 7,104 participants with CVD at baseline and 1,653 participants diagnosed with CVD before dietary assessment were excluded, resulting in a final analytic cohort of 164,111 individuals (Supplementary Fig. S1).

#### 2.1.2 Measurement of dietary Mn

Dietary data were collected using a valid web-based 24-h dietary instrument (Oxford WebQ) that was designed for use in large population studies [15]. In this study, we retained participants with at least one dietary assessment for analysis, and for participants that provided more than one 24-h recall, we calculated the average dietary Mn intake data (Supplementary Methods).

#### 2.1.3 Other covariates

Baseline information on socio-demographic characteristics, lifestyles factors including smoking status, alcohol intake, and physical activity, diet, and medical history were collected using touchscreen questionnaire. Dietary variables like energy intake, diet quality as assessed by the Alternative Healthy Eating Index 2010 (AHEI-2010), and dietary antioxidant intake were computed from dietary data. Additional information on covariates is found in Supplementary Methods.

#### 2.1.4 Outcome ascertainment

The outcome variables in this study were T2D incidence, total CVD, and CVD mortality. Detailed information on outcome ascertainment is provided in Supplementary Methods.

#### 2.1.5 Statistical analysis

Baseline characteristics of participants are presented by five quintiles of dietary Mn intake. While categorical variables are presented as frequencies (percentages), continuous variables are presented as means (standard deviation) if normally distributed and median (IQR) if not-normally distributed.

We employed Cox proportional hazards regression models to estimate the hazard ratios (HRs) and 95% CIs for risk of T2D (T2D analytic cohort) and for risk of total CVD and CVD mortality (CVD analytic cohort). We categorized the energy-adjusted Mn intake into five quintiles and considered the lowest category (Q1) as reference in our analyses. We fitted three models in both analytic cohorts. Models were adjusted for covariates. In addition, we assessed the proportional hazards assumption using Schoenfeld residuals.

Sensitivity analyses and restricted cubic splines (RCS) analyses for modelling non-linear dose-response associations were also conducted [16]. We modeled Mn intake using RCS with five knots placed at the 5th, 27.5th, 50th, 72.5th, and 95th percentiles of the intake distribution. Nonlinearity was assessed using Wald tests, with p-values for nonlinearity (PNL) and linear trend (PLIN) reported. Pointwise 95% confidence intervals were calculated for spline estimates, and marginal effects were derived at selected intake levels. We also determined E-values for the Q5 vs Q1 contrast to evaluate robustness to unmeasured confounding.

In another sensitivity analysis, we restricted the sample to participants with ≥2, ≥3, ≥4, and ≥5 repeated 24-h dietary recalls assessing whether the association between Mn intake and outcomes was affected by exposure measurement frequency. Moreover, competing risks were addressed using Fine-Gray sub-distribution hazard models for CVD incidence and cause-specific Cox models for CVD mortality.

Statistical data analysis was performed using R software version 3·6·1 and statistical significance was declared at a P value of <0·05. More detail is provided in Supplementary Methods.

#### 2.1.6 Ethics statement

The UK Biobank study was approved in 2011 by the North West Multi-centre Research Ethics Committee (MREC) (Reference number: 11/NW/0382) as a Research Tissue Bank (RTB) approval. All participants provided written informed consent.

### 2.2 Meta-analysis of prospective cohorts

#### 2.2.1 Search strategy

The procedures for this meta-analysis have been registered at PROSPERO (CRD42024628509). We performed a systematic search of the databases PubMed, Web of Science, Embase, and Scopus for prospective cohort studies published through Dec 31, 2024. Detailed information on the keywords and database search is provided in Supplementary Methods.

#### 2.2.2 Study selection and data extraction

We included studies that met the following five criteria: (1) Prospective study design; (2) Dietary intake of manganese as exposure (3) The outcome was T2D, incidence and/or mortality due to CVD (including coronary heart disease/ischemic heart disease, and/or stroke); and (4) The authors provided risk estimates accompanied by 95% confidence intervals (95% CI); (5) The number of participants exposed and the number of events in different categories of Mn intake were indicated.

From each included study, we extracted information on: the first author and year of the publication, the average Mn intake of each quartile/quintile, sex, the number of participants in each quartile/quintile, number of cases in each category of Mn intake, effect estimates in the form of relative risk (RR), and its lower and upper 95% CI, the standard error, follow-up period, dietary assessment method, and geographical information.

Dietary Mn intake was harmonized across studies using mg/day as the common unit. Most cohorts assessed intake via FFQs, while the UK Biobank used repeated 24-h recalls. For studies reporting intake categories, we imputed midpoints based on reported ranges. Nutrient values were mapped using study-specific food composition databases, and dose-scaling assumptions were applied to approximate continuous exposure. Details of the study selection and data extraction procedures are provided in Supplementary Methods.

#### 2.2.3 Quality assessment

The quality of the included studies was assessed using the Newcastle-Ottawa Scale (NOS) [17]. This scale assigns 4 points for the selection of participants and ascertainment of exposure, 2 points for comparability of studies based on design or analysis, and 3 points for assessment of outcomes and adequate duration of follow-up, totaling a maximum of 9. In this study, the overall quality of the studies was good except for one study that scored fair (Supplementary Table S2). Additionally, risk of bias for each included study was evaluated using the Risk Of Bias In Non-randomized Studies - of Exposures (ROBINS-E) assessment tool, which contains seven domains of bias due to (1) confounding, (2) exposure assessment, (3) selection of participants into study (or into the analysis), (4) misclassification of exposure during follow-up, (5) missing data, (6) measurement of the outcome, and (7) selective reporting of the results [18]. Each domain was rated as low risk, some concerns, high risk, or very high risk, based on which a final judgment on risk of bias was made for each study. All five studies included in this meta-analysis were judged to have “some concerns” risk of bias overall, primarily due to limitations in confounding control and exposure assessment (Supplementary Table S3). In line with this, certainty of evidence for each outcome was assessed using the Grading of Recommendations Assessment, Development and Evaluation (GRADE) framework [19]. It was rated as low for both T2D and CVD outcomes, primarily due to moderate risk of bias and imprecision (Supplementary Table S4).

#### 2.2.4 Dose-response meta-analysis

Separate analyses for T2D and for total CVD (incidence or mortality) were conducted. From each included study, we extracted effect estimates reported as RR, HR, or odds ratio (OR), with their 95% CIs. These effect estimates were combined based on the evidence that when the incidence of outcomes is below 10%, RR, OR and HR are approximately equivalent [20,21]. To assess the robustness of HRs, RRs, and ORs under this rare outcome assumption, we conducted a sensitivity analysis excluding the only study that reported ORs. In cases where studies divided data by sex and/or cohort subgroup, they were regarded as two distinct reports.

We estimated a pooled risk with 95% CI for a 1 mg/day increase in original dietary Mn intake for the included studies using the restricted maximum likelihood (REML) approach to a summarized dose-response data as proposed by Greenland and Longnecker and Orsini et al. [22,23]. To better evaluate the robustness and generalizability of any observed associations, we conducted a series of sensitivity analyses including meta-regression, leave-one-out analysis, and prediction intervals.

Although the methods could be underpowered with fewer than 10 studies, we conducted a qualitative assessment of potential publication bias using funnel plots and Egger’s regression test [24,25]. Asymmetry in the funnel plot and a p-value < 0.10 from Egger’s test were considered indicative of small-study effects. A trim-and-fill analysis was also performed to detect and adjust for any potential publication bias. More information on the dose-response meta-analysis procedures is provided in Supplementary Methods. The dose-response meta-analysis was conducted using the dosr*esmeta* package in R and p-value less than 0·05 was considered statistically significant.

## 3 Results

### 3.1 Baseline characteristics of participants in UK Biobank

A total of 165,194 participants were included in the T2D analysis. During a mean follow-up of 10·6 years (1,784,556 person-years), 5,198 (3·1%) participants developed T2D. The incidence of T2D decreased across the quintiles, from 3·9% in the lowest Mn intake group (Q1) to 2·8% in the highest (Q5). The mean (±SD) Mn intake ranged from 2·36 ± 0.51 mg in Q1 to 6·50 ± 1·09 mg in Q5. The mean age slightly increased across quintiles, from 54·6 ± 8·1 years in Q1 to 56·2 ± 7·9 years in Q5. The CVD analytic cohort included 164,111 participants, of which 11,941 (7·2%) developed CVD over a mean follow-up of 10·8 years (1,744,393 person-years). The incidence of CVD showed a slight decreasing trend across quintiles, from 8% in Q1 to 7% in Q5. The mean (±SD) Mn intake ranged from 2·36 ± 0·51 mg in Q1 to 6·50 ± 1·09 mg in Q5 (Table 1).

**Table 1:** Baseline characteristics of participants in T2D and CVD analytic cohorts by Manganese intake quintiles.

Variable	T2D analytical cohort						CVD analytical cohort					
	Q1	Q2	Q3	Q4	Q5	p-value	Q1	Q2	Q3	Q4	Q5	p-value
n	33039	33039	33038	33039	33039	NA	32857	32818	32788	32828	32820	<0.001
Mn, mean (SD)	2.36 (0.51)	3.40 (0.22)	4.12 (0.20)	4.90 (0.26)	6.50 (1.09)	<0.001	2.36 (0.51)	3.40 (0.22)	4.12 (0.20)	4.90 (0.26)	6.50 (1.09)	<0.001
Age (mean (SD))	54.60 (8.07)	55.59 (7.99)	56.09 (7.87)	56.34 (7.83)	56.20 (7.93)	<0.001	54.47 (8.03)	55.44 (7.97)	55.96 (7.86)	56.18 (7.82)	56.07 (7.93)	<0.001
Male, %	14015 (42.4)	13855 (41.9)	14420 (43.6)	15411 (46.6)	18024 (54.6)	<0.001	13740 (41.8)	13595 (41.4)	14117 (43.1)	15076 (45.9)	17737 (54.0)	<0.001
Ethnicity, %						<0.001						<0.001
Asian	704 (2.1)	527 (1.6)	448 (1.4)	390 (1.2)	478 (1.4)		760 (2.3)	559 (1.7)	478 (1.5)	425 (1.3)	516 (1.6)	
Black	679 (2.1)	342 (1.0)	252 (0.8)	243 (0.7)	304 (0.9)		714 (2.2)	370 (1.1)	266 (0.8)	263 (0.8)	321 (1.0)	
Mixed	237 (0.7)	177 (0.5)	159 (0.5)	160 (0.5)	204 (0.6)		246 (0.7)	180 (0.5)	164 (0.5)	164 (0.5)	207 (0.6)	
White	31021 (93.9)	31691 (95.9)	31922 (96.6)	31956 (96.7)	31720 (96.0)		30707 (93.5)	31377 (95.7)	31603 (96.4)	31762 (96.5)	31400 (95.7)	
Other ethnic group	398 (1.2)	302 (0.9)	257 (0.8)	290 (0.9)	333 (1.0)		401 (1.2)	313 (1.0)	258 (0.8)	288 (0.9)	353 (1.1)	
BMI, mean (SD)	27.49 (4.73)	26.98 (4.42)	26.68 (4.35)	26.31 (4.22)	25.98 (4.19)	<0.001	27.60 (4.85)	27.07 (4.54)	26.77 (4.49)	26.37 (4.31)	26.05 (4.31)	<0.001
Waist circumference, mean (SD)	–	–	–	–	–	NA	90.06 (13.67)	88.87 (13.17)	88.35 (13.07)	87.84 (12.74)	87.89 (12.70)	<0.001
TDI, mean (SD)	−1.34 (3.01)	−1.69 (2.80)	−1.73 (2.79)	−1.75 (2.76)	−1.63 (2.84)	<0.001	−1.32 (3.02)	−1.68 (2.80)	−1.72 (2.80)	−1.74 (2.76)	−1.61 (2.85)	<0.001
Lower education level, %	21011 (63.6)	19341 (58.5)	18070 (54.7)	16849 (51.0)	15609 (47.2)	<0.001	20859 (63.5)	19138 (58.3)	17902 (54.6)	16649 (50.7)	15395 (46.9)	<0.001
Family history of CVD, %	–	–	–	–	–	NA	8867 (27.0)	9213 (28.1)	9377 (28.6)	9481 (28.9)	9405 (28.7)	<0.001
Family history of T2D, %	6095 (18.4)	5765 (17.4)	5597 (16.9)	5537 (16.8)	5217 (15.8)	<0.001	–	–	–	–	–	NA
Baseline diabetes, %	–	–	–	–	–	NA	1273 (3.9)	1199 (3.7)	1130 (3.4)	1102 (3.4)	1136 (3.5)	0.003
Baseline CVD, %	1276 (3.9)	1175 (3.6)	1127 (3.4)	1236 (3.7)	1199 (3.6)	0.024	–	–	–	–	–	NA
Baseline hypertension, %	7208 (21.8)	6861 (20.8)	6817 (20.6)	6657 (20.1)	6239 (18.9)	<0.001	7922 (24.1)	7438 (22.7)	7402 (22.6)	7229 (22.0)	6802 (20.7)	<0.001
Smoking, %						<0.001						<0.001
Current	3994 (12.1)	2716 (8.2)	2166 (6.6)	1938 (5.9)	1823 (5.5)		3947 (12.0)	2659 (8.1)	2171 (6.6)	1937 (5.9)	1847 (5.6)	
Never	17676 (53.5)	18526 (56.1)	19076 (57.7)	19517 (59.1)	19713 (59.7)		17728 (54.0)	18544 (56.5)	19002 (58.0)	19501 (59.4)	19610 (59.8)	
Previous	11369 (34.4)	11797 (35.7)	11796 (35.7)	11584 (35.1)	11503 (34.8)		11182 (34.0)	11615 (35.4)	11615 (35.4)	11390 (34.7)	11363 (34.6)	
Alcohol Drinking, %						<0.001						<0.001
Current	30862 (93.4)	31240 (94.6)	31359 (94.9)	31227 (94.5)	30969 (93.7)		30638 (93.2)	31008 (94.5)	31078 (94.8)	310008 (94.5)	30755 (93.7)	
Never	1166 (3.5)	960 (2.9)	884 (2.7)	916 (2.8)	1003 (3.0)		1193 (3.6)	984 (3.0)	913 (2.8)	921 (2.8)	999 (3.0)	
Previous	1011 (3.1)	839 (2.5)	795 (2.4)	896 (2.7)	1067 (3.2)		1026 (3.1)	826 (2.5)	797 (2.4)	899 (2.7)	1066 (3.2)	
IPAQ, mean (SD)	1.13 (0.75)	1.17 (0.74)	1.21 (0.72)	1.25 (0.71)	1.32 (0.70)	<0.001	1.13 (0.75)	1.17 (0.74)	1.20 (0.73)	1.25 (0.71)	1.32 (0.70)	<0.001
Energy intake, mean (SD)	1647.5 (454.8)	1911.4 (454.2)	2059.3 (465.5)	2213.4 (490.9)	2538.7 (603.1)	<0.001	1645.1 (455.2)	1949.5 (454.9)	2057.6 (466.8)	2211.3 (490.5)	2539.6 (605.4)	<0.001
AHEI2010, mean (SD)	42.45 (11.14)	50.12 (11.19)	54.62 (11.35)	58.11 (11.48)	61.76 (12.00)	<0.001	45.9 (12.1)	50.9 (12.4)	54.1 (12.4)	56.6 (12.4)	59.7 (12.6)	<0.001
DAI, n (%)												
q1	11505 (34.8)	7212 (21.8)	5736 (17.4)	4597 (13.9)	3680 (11.1)	<0.001	8992 (27.4)	6933 (21.1)	6160 (18.8)	5453 (16.6)	4948 (15.1)	<0.001
q2	7370 (22.3)	7438 (22.5)	6922 (21.0)	6175 (18.7)	4972 (15.0)		7847 (23.9)	7169 (21.8)	6609 (20.2)	6140 (18.7	4880 (14.9)	
q3	5864 (17.7)	7059 (21.4)	7144 (21.6)	6939 (21.0)	6124 (18.5)		6569 (20.0)	7094 (21.6)	6891 (21.0)	6668 (20.3)	5643 (17.2)	
q4	4664 (14.1)	6341 (19.2)	7171 (21.7)	7639 (23.1)	7382 (22.3)		5464 (16.6)	6496 (19.8)	7035 (21.5)	7160 (21.8)	6821 (20.8)	
q5	3636 (11.0)	4989 (15.1)	6065 (18.4)	7689 (23.3)	10881 (32)		3985 (12.1)	5126 (15.6)	6093 (18.6)	7407 (22.6)	10528 (32.1)	
Multivitamin	–	–	–	–	–	NA	5517 (16.8)	5976 (18.2)	6189 (18.9)	6393 (19.4)	7003 (21.3)	<0.001
Follow up years, mean (SD)	10.86 (2.02)	10.78 (1.86)	10.75 (1.80)	10.77 (1.75)	10.86 (1.82)	<0.001	10.70 (2.27)	10.62 (2.12)	10.60 (2.06)	10.58 (2.06)	10.65 (2.15)	<0.001

Abbreviations: AHEI-2010; Alternative Healthy Eating Index-2010, BMI; Body mass index, CVD; Cardiovascular diseases, DAI; Dietary antioxidant index, IPAQ; International Physical Activity Questionnaire, Mn; Manganese, SD; Standard deviation, TDI; Townsend deprivation index, T2D; Type 2 diabetes, NA; Not applicable.

### 3.2 Mn intake and T2D incidence in UK Biobank

In the overall sample (n = 165,194), the fully adjusted model (Model 3) showed that high (Q5) energy-adjusted Mn intake is not significantly associated with T2D risk (HR for Q5 0·91; 95% CI 0·82, 1.01, *p_trend_* = 0·07)), compared to the reference Mn intake, Q1 (Table 2). No significant interaction was observed between sex and Mn intake (p = 0.66), suggesting no evidence of sex-based effect modification (Supplementary Table S5). Similarly, subgroup analyses showed that there was no significant association in men (n = 75,725) or women (n = 89,469) participants (Table 3).

**Table 2:** Hazard ratios for the association of energy-adjusted dietary Mn intake with T2D incidence, total CVD, and CVD mortality.

Cox-PH (HR)	Q1	Q2	Q3	Q4	Q5	Continuous	P for trend
**T2D (*N = 165,194)***							
Incident cases	1,278	1,072	1023	904	921	NA	NA
Person-years	358,773	356,078	355,159	355,899	358,650	NA	NA
Model 1	ref	0.75 (0.69,0.81)	0.66 (0.61,0.72)	0.6 (0.55,0.66)	0.55 (0.50,0.60)	0.84 (0.82,0.86)	<0.01
Model 2	ref	0.83 (0.77,0.90)	0.78 (0.72,0.85)	0.74 (0.68,0.81)	0.70 (0.64,0.78)	0.91 (0.88,0.93)	<0.01
Model 3	ref	0.93 (0.85,1.01)	0.91 (0.83,1.00)	0.92 (0.83,1.01)	0.91 (0.82,1.01)	0.98 (0.95,1.00)	0.07
**Total CVD (*N = 164,111)***							
Incident cases	2,508	2,315	2,286	2,374	2,458	NA	NA
Person-years	351,635	348,425	347,419	347,260	349,656	NA	NA
Model 1	ref	0.87 (0.82, 0.92)	0.81 (0.77, 0.86)	0.84 (0.79, 0.89)	0.78 (0.74, 0.83)	0.94 (0.93, 0.95)	<0.01
Model 2	ref	0.91 (0.86, 0.96)	0.91 (0.86, 0.96)	0.87 (0.82, 0.92)	0.88 (0.83, 0.94)	0.97 (0.96, 0.99)	<0.01
Model 3	ref	0.95 (0.90, 1.01)	0.94 (0.88, 0.99)	1.01 (0.95, 1.08)	0.99 (0.92, 1.05)	1.00 (0.99, 1.02)	0.61
**CVD mortality** (**N = 164,111)**							
Cases	118	112	107	90	115	NA	NA
Person-years	351,635	348,425	347,419	347,260	349,656	NA	NA
Model 1	ref	0.49 (0.37,0.65)	0.70 (0.55, 0.90)	0.64 (0.50,0.82)	0.64 (0.50,0.82)	0.90 (0.84,0.97)	<0.01
Model 2	ref	0.51 (0.39,0.68)	0.75 (0.58,0.97)	0.70 (0.53,0.92)	0.73 (0.55,0.97)	0.94 (0.87,1.02)	0.12
Model 3	ref	0.55 (0.42,0.74)	0.83 (0.64,1.08)	0.81 (0.61,1.07)	0.85 (0.64,1.13)	0.98 (0.91,1.06)	0.66

Model 1 was adjusted for age, sex and race/ethnicity; Model 2 was further adjusted for dietary energy, AHEI-2010, and DAI; Model 3 (outcome: T2D incidence) was further adjusted by socioeconomic status, education, smoking status, alcohol drinking, physical activity measured by International Physical Activity Questionnaire (IPAQ), family T2D history, hypertension at baseline, CVD at baseline, and body mass index (BMI). Model 3 (outcome: total CVD and mortality) was further adjusted for socioeconomic status, education, smoking status, alcohol drinking, physical activity measured by IPAQ, family CVD history, hypertension at baseline, diabetes at baseline, and BMI.

Abbreviations: AHEI-2010; Alternative Healthy Eating Index-2010, Cox-PH (HR); Cox proportional hazard (hazard ratio), CVD; Cardiovascular diseases, DAI; Dietary antioxidant intake, ref; reference group, Q; quintile, T2D; Type 2 diabetes, NA; Not applicable.

**Table 3:** Sex-specific hazard ratios for the association of energy-adjusted dietary Mn intake with T2D incidence, total CVD, and CVD mortality.

		Men						Women					
Cox-PH (HR)	Q1	Q2	Q3	Q4	Q5	Continuous	P for trend	Q2	Q3	Q4	Q5	Continuous	P for trend
**T2D incidence *(N = 75,725)***								**T2D incidence (N = 89,469)**					
Model 1	ref	0.77 (0.70,0.86)	0.69 (0.62,0.77)	0.60 (0.54,0.67)	0.53 (0.47,0.59)	0.85 (0.83,0.87)	<0.01	0.74 (0.66,0.84)	0.65 (0.57,0.75)	0.58 (0.51,0.67)	0.56 (0.49,0.65)	0.83 (0.79,0.86)	<0.01
Model 2	ref	0.78 (0.70,0.86)	0.79 (0.71,0.89)	0.71 (0.63,0.80)	0.66 (0.59,0.75)	0.90 (0.87,0.93)	<0.01	0.91 (0.80,1.04)	0.77 (0.67,0.89)	0.79 (0.68,0.91)	0.77(0.65,0.90)	0.91 (0.87,0.95)	<0.01
Model 3	ref	0.87 (0.78,0.98)	0.92 (0.81,1.01)	0.88 (0.78,1.01)	0.90 (0.79,1.03)	0.98 (0.94,1.01)	0.17	1.02 (0.88,1.17)	0.91 (0.78,1.07)	0.96 (0.82,1.13)	0.92 (0.78,1.10)	0.97 (0.93,1.02)	0.21
**Total CVD (N = 74,265)**								**Total CVD (N = 89,846)**					
Model 1	ref	0.88 (0.82, 0.95)	0.80 (0.75, 0.86)	0.80 (0.74, 0.86)	0.77 (0.72, 0.83)	0.94 (0.92, 0.95)	<0.01	0.87 (0.79, 0.96)	0.80 (0.73, 0.88)	0.92 (0.83, 1.00)	0.82 (0.74, 0.90)	0.95 (0.93, 0.98)	<0.01
Model 2	ref	0.91 (0.84, 0.97)	0.84 (0.78, 0.91)	0.85 (0.79, 0.92)	0.84 (0.78, 0.91)	0.96 (0.94, 0.98)	<0.01	0.93 (0.84, 1.02)	0.89 (0.81, 0.99)	1.05 (0.95, 1.16)	0.97 (0.87, 1.08)	1.00 (0.97, 1.03)	0.91
Model 3	ref	0.95 (0.89, 1.02)	0.91 (0.84, 0.98)	0.94 (0.87, 1.02)	0.95 (0.88, 1.04)	0.99 (0.97, 1.01)	0.39	0.98 (0.89, 1.09)	0.97 (0.88, 1.08)	1.16 (1.05, 1.28)	1.08 (0.97, 1.20)	1.03 (1.00, 1.06)	0.04
**CVD mortality (N = 74,265)**								**CVD mortality (N = 89,846)**					
Model 1	ref	0.59 (0.44,0.81)	0.60 (0.44,0.81)	0.69 (0.52,0.91)	0.62 (0.46,0.83)	0.90 (0.83,0.97)	<0.01	0.52 (0.29,0.94)	0.73 (0.44,1.24)	0.67 (0.39,1.14)	0.68 (0.40,1.15)	0.94 (0.80,1.10)	0.43
Model 2	ref	0.59 (0.43,0.80)	0.59 (0.44,0.79)	0.68 (0.51,0.90)	0.61 (0.46,0.82)	0.90 (0.83,0.97)	<0.01	0.53 (0.29,0.94)	0.74 (0.44,1.24)	0.67 (0.39,1.14)	0.68 (0.40,1.14)	0.94 (0.80,1.10)	0.42
Model 3	ref	0.64 (0.47,0.88)	0.68 (0.49,0.93)	0.83 (0.60,1.14)	0.77 (0.55,1.09)	0.96 (0.87,1.04)	0.31	0.65 (0.36,1.18)	1.00 (0.58,1.74)	0.98 (0.55,1.739)	1.07 (0.59,1.94)	1.09 (0.92,1.29)	0.33

Model 1 was adjusted for age and race/ethnicity; Model 2 was further adjusted for dietary energy, AHEI-2010, and DAI; Model 3 (outcome: T2D incidence) was further adjusted for socioeconomic status, education, smoking status, alcohol drinking, physical activity measured by International Physical Activity Questionnaire (IPAQ), family T2D history, hypertension at baseline, CVD at baseline, and body mass index (BMI). Model 3 (outcome: total CVD and mortality) was further adjusted for socioeconomic status, education, smoking status, alcohol drinking, physical activity measured by IPAQ, family CVD history, hypertension at baseline, diabetes at baseline, and BMI.

Abbreviations: AHEI-2010; Alternative Healthy Eating Index-2010, Cox-PH (HR); Cox proportional hazard (hazard ratio), CVD; Cardiovascular diseases, DAI; Dietary antioxidant intake, ref; reference group, Q; quintile, T2D; Type 2 diabetes, NA; Not applicable.

### 3.3 Mn intake and total CVD in UK Biobank

**Fig. 1:**
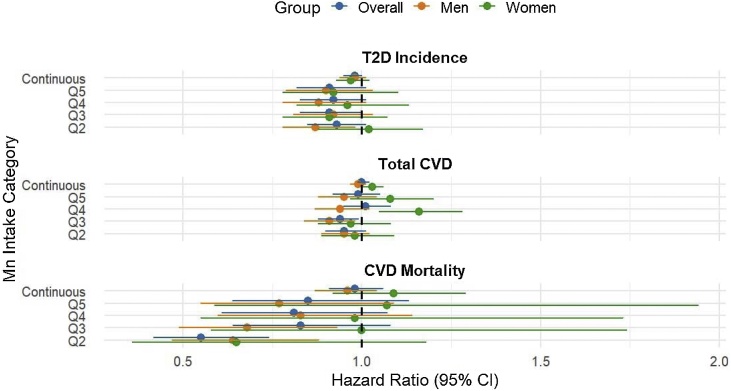
Forest plot of HRs (95% CI) of T2D, total CVD, and CVD mortality by quintiles of energy-adjusted Mn intake. This figure shows hazard ratios (HRs) and 95% confidence intervals (CIs) for associations between energy-adjusted manganese (Mn) intake quintiles and risks of type 2 diabetes (T2D), total cardiovascular disease (CVD), and CVD mortality. Abbreviations: CI, Confidence interval; CVD, cardiovascular disease; HR, Hazard ratio; Mn; Manganese; T2D, Type 2 diabetes.

In the overall sample (n = 164,111), Model 3 did not show a significant association between Q5 energy-adjusted Mn intake and CVD risk (Adjusted HR 0·99; 95% CI 0·92, 1.05) (Table 2). Likewise, subgroup analyses did not indicate significant association between Mn intake and CVD incidence in either the men (n = 74,265) or women (n = 89,846) samples (Table 3). Moreover, energy-adjusted Mn intake showed no significant association with risk of CVD mortality (Table 2). Fig. 1 shows forest plots of HRs of T2D, total CVD, and CVD mortality by Mn intake categories. Regarding CVD subtypes, Q5 energy-adjusted dietary Mn intake did not exhibit any significant association with the risk of MI (HR 0·96; 95% CI 0·84, 1.09), CHD (HR 0·98; 95% CI 0·91, 1.05), or HF (HR 0·97; 95% CI 0·85, 1.10) (Supplementary Table S6).

### 3.4 Sensitivity analysis in UK Biobank

In Model 3, compared to Q1, Q5 original Mn intake was not significantly associated with risk of T2D (Adjusted HR 0·93; 95% CI 0·82, 1.05, p_trend_ = 0.07), consistent with the non-significant link between energy-adjusted Mn intake and T2D risk. Similarly, the sensitivity analysis did not reveal a significant relationship between higher original (non-energy-adjusted) dietary Mn intake and reduced risk of total CVD (Adjusted HR for Q5 0·99; 95% CI 0·92, 1.06, p_trend_ = 0.61) (Supplementary Table S7).

Another sensitivity analysis, which focused on participants in our sample who had completed ≥2, ≥3, ≥4, and ≥5 dietary assessments demonstrated consistent associations in these subgroups, suggesting that misclassification due to intake variability had limited impact on the observed estimates (Supplementary Table S8).

In our sensitivity analysis of proportional hazard (PH) assumptions, there were no significant global violations or violations for the exposure of interest (p = 0.79 for T2D & p = 0.92 for CVD), except for mild deviations in some covariates (e.g., physical activity, education) that did not materially affect estimates (Supplementary Table S9). To avoid reverse causality, we run a lagged analysis by excluding cases identified in the first two years of follow up, but this did not reveal any significant differences from the primary findings (Supplementary Table S10).

In the competing risks regression, although there was no statistically significant association with CVD incidence when accounting for competing risk of non-CVD death, there were significant inverse associations between energy-adjusted Mn intake and CVD mortality (HR for Q2 0.56; 95% CI 0.42, 0.74), MI (HR for Q3 0.83; 95% CI 0.74, 0.94), and CHD (HR for Q3 0.93; 95% CI 0.87, 0.99) (Supplementary Table S11).

### 3.5 Dose-response analysis in UK Biobank

**Fig. 2:**
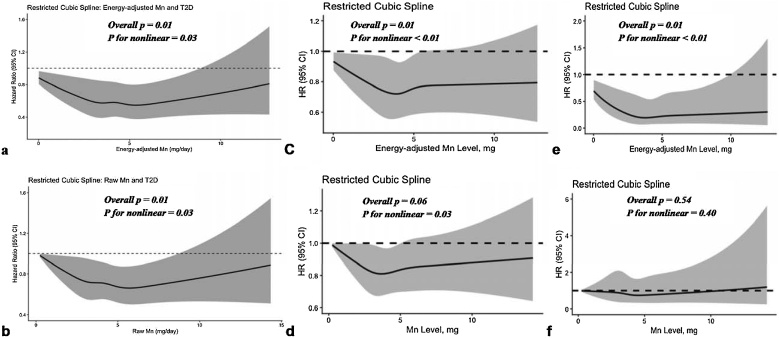
Restricted cubic spline analysis of energy-adjusted and original dietary manganese intake with hazard ratio of T2D incidence (a & b), Total CVD (c & d), and CVD mortality (e & f) in UK Biobank population. This figure shows restricted cubic spline analysis for non-linear associations of energy-adjusted and original dietary Mn intake with HRs of T2D incidence (a & b), Total CVD (c & d), and CVD mortality (e & f) in UK Biobank cohort.

Our RCS analyses revealed nonlinear inverse associations between Mn intake and T2D, total CVD, and CVD mortality risk. For T2D, the knot locations were at 2.06, 3.31, 4.12, 5.00, and 6.92 mg/day of Mn intake. Both energy-adjusted and original dietary Mn intake showed a non-linear inverse relationship with T2D risk (*PNL* = 0.03) with a borderline linear trend (*PLIN* = 0.07), as the curve flattened at ∼5 mg/day (Fig. 2a and b). The Q5 vs Q1 contrast yielded RR = 0.91 (95% CI 0.82, 1.01), with an E‑value of 1.44 (Supplementary Table S13).

For total CVD incidence, spline models displayed significant nonlinearity (*PNL < 0.01*) but no linear trend (*PLIN = 0.61*) (Fig. 2c and d). The Q5 vs Q1 contrast yielded RR = 0.99, 95% CI 0.92, 1.05), with an E‑value of 1.13 (Supplementary Table S12). Similarly, the risk of CVD mortality decreased nonlinearly with increasing dietary Mn intake (PNL = 0·01) with no linear trend (*PLIN* = 0.66) (Fig. 2e and f).

For all outcomes, because the 95% CI included the null (HR ≈ 1), the E‑value for the CI limit was 1.00. This indicates that no residual confounding would be required to shift the confidence interval to include the null, consistent with the non‑significant result.

### 3.6 Meta-analysis of prospective cohorts

Our initial database search identified a total of 2,566 articles. Out of these, 1,800 articles were deemed potentially relevant for further review. We excluded 1,796 articles for several reasons including irrelevant topics, irrelevant exposures, irrelevant outcomes, review papers, etc. (Supplementary Fig. S2). Ultimately, five studies (three for T2D and two for CVD) were included [[10], [11], [12], [13], [14]]. The present UKB study was also added as the sixth study.

#### 3.6.1 Study characteristics

Supplementary Tables S13 and S14 contain the baseline characteristics of the studies that are part of this systematic review and dose-response meta-analysis. The sample size of the studies ranged from 10,483 to 84,285. Only one of the six studies included one sex (women) only [10]. All studies but the UKB used food frequency questionnaires for dietary assessment [[10], [11], [12], [13], [14]].

#### 3.6.2 Dietary Mn and T2D risk

In this meta-analysis of 279,824 individuals, the overall model was statistically significant (p < 0·01). The pooled estimate indicated that a 1 mg/day increment per day in Mn intake was associated with 4% decrease in T2D risk (pooled RR 0·96; 95% CI 0·94, 0·99), with heterogeneity (I^2^ = 69.0% and Cochran's Q-test (Q = 19.3, df = 6, p = 0·03). Besides, the RCS model showed the existence of non-linearity in the relationship (p < 0·001) (Supplementary Fig. S3).

#### 3.6.3 Sensitivity analysis

Our sensitivity analyses excluding a study thar reported ORs [12] generated a pooled RR of 0.93 (95% CI 0.90, 0.97), consistent with the primary analysis. Besides, our leave-one-out sensitivity analysis showed that the pooled log relative risk ranged from −0·12 to −0·19 when each study was omitted in turn, with all estimates remaining statistically significant at p < 0·01, except when Gong et al. [10] was removed, in which statistical significance was reached at p = 0.02, with heterogeneity (I²) of 32.7% to 60.3%, and the leave-one-out plot showed that the pooled logRR remained consistently negative, indicating the robustness of the primary findings (Supplementary Fig. S4).

Additionally, our meta-regression model using dietary assessment method, geographic region, sex, and follow-up duration as moderators explained all between-study variability (I² = 0%, R² = 100.00 %), with no residual heterogeneity (QE p = 0.38). While none of these moderators reached statistical significance (all p > 0.05), they collectively contributed to the observed heterogeneity (QM = 18.6, df = 5, p < 0.01) (Supplementary Table S15). Moreover, the 95% prediction interval ranged from 0.92 to 1.02 (RR per 1 mg/day Mn intake), suggesting that future studies may likely observe a similar association.

Furthermore, Egger’s regression test for funnel plot asymmetry showed no evidence of publication bias (z = −0.07, p = 0.94), with a symmetrical funnel plot indicating no small-study effects (Supplementary Fig. S5). Likewise, our trim-and-fill analysis indicated no missing studies and produced an adjusted effect estimate (RR 0.96; 95% CI 0.94, 0.99) similar to the original finding, suggesting minimal risk of publication bias.

#### 3.6.4 Dietary Mn and total CVD risk

In this meta-analysis of 284,846 individuals, the overall model was not statistically significant (pooled RR 0·99; 95% CI 0·98, 1·02, p = 0·22) with no residual heterogeneity (I^2^ = 0·0%, Cochran's Q-test p = 0·78). Similarly, sex-specific analysis did not indicate statistically significant associations in women (pooled RR 0·98; 95% CI 0·97, 1·02, p = 0·29) or men (pooled RR 0·99; 95% CI 0·98, 1·02, p = 0·25). There was no non-linear association between Mn intake and total CVD (Supplementary Fig. S6).

## 4 Discussion

### 4.1 Summary of main findings

In this study, we examined the associations between dietary Mn and the risk of T2D and CVD using a large prospective cohort of >160,000 UK Biobank participants. Our findings were further substantiated by a meta-analysis involving >270,000 individuals from multiple cohorts including UK Biobank. While our UK Biobank analysis did not reveal a significant association between Mn intake and T2D risk, our dose-response meta-analysis indicated a 4% decrease in T2D risk with each 1 mg/day increment in Mn intake, with potential non-linearity. Moreover, Mn intake was not significantly linked to a lower risk of total CVD or CVD mortality, in either the UK Biobank analysis or the meta-analysis.

### 4.2 Interpretation of findings

Regarding T2D incidence, our UK Biobank findings did not align with previous studies that reported protective role of dietary Mn against T2D risk. Our analyses adjusted for several dietary factors including AHEI-2010 and DAI as measures of diet quality. However, prior studies have reported significant inverse associations even after adjusting for dietary quality. For instance, Du et al. observed a protective effect of Mn intake in two large Chinese cohorts, despite controlling for dietary factors including total antioxidant capacity (TAC) as proxy measure of diet quality [11]. The protective effect of Mn in T2D could be explained by several mechanistic pathways. Studies have shown the association between Mn homeostasis in the liver and insulin sensitivity [26], its crucial role as a cofactor for superoxide dismutase in reducing oxidative stress [27], and its involvement in glucose and lipid metabolism, as well as its significance for mitochondrial function [9]. Besides, in mice, Mn supplementation resulted in improved glucose tolerance, insulin secretion, and increased mitochondrial function in pancreatic islets under high-fat diet [28].

In the overall UK Biobank sample, there was no significant association between Mn intake and CVD risk or CVD mortality. Likewise, our dose-response meta-analysis did not detect any statistically significant links between Mn intake and CVD risk. Similarly, Yazdanpanah et al., found no significant association between Mn intake (Q5) and CVD mortality (HR 1·07; 95% CI 0·87, 1·20, p*_trend_* = 0·29) [14]. In contrast, higher Mn intake was related to reduced CVD mortality in Japanese adults, with stronger associations observed in postmenopausal women [13]. These discrepancies may be due to variations in study populations, dietary assessment methods, and residual confounding. This underscores the complexity of dietary Mn’s role in the development of CVD and emphasizes the need for further research. Mechanistic and longitudinal studies are particularly necessary to gain a more nuanced understanding of the relationship between dietary Mn and CVD risk.

### 4.3 Dose-response relationship and the implication for dietary recommendations

From the dose-response meta-analysis of UK Biobank data and the available prospective cohorts, we observed a non-linear inverse relationship for T2D, with risk reduction plateauing when Mn intake reached 5 mg/day. These findings may have important implications for dietary guidelines and recommendations. Nonetheless, these results are from observational data, which may be influenced by residual confounding, and warrant cautious interpretation and replication in intervention studies. Current guidelines suggest that women should have a daily intake of 1.8 mg of Mn, while men are advised to consume 2.3 mg each day to ensure adequate intake [6]. Our findings showed that Mn may reduce T2D risk even at levels higher than these current guidelines and highlight the need for further research, particularly trials, to clarify its role in chronic disease risk reduction. Given that Mn is commonly found in foods such as whole grains, green leafy vegetables, nuts and seeds, legumes, coffee and tea, promoting a Mn-rich diet could aid in the prevention of T2D [29]. On the other hand, while observed dietary Mn intakes in our study are generally well below the tolerable upper intake level (UL), which is 11 mg/day for adults 19 years of age or older [30], and not much evidence shows Mn toxicity from high dietary Mn intakes whatsoever [31], it is important to recognize that excessive Mn intake, particularly from occupational or environmental exposures, has been linked to neurotoxicity in vulnerable populations [32].

The sensitivity analyses for our dose-response meta-analysis collectively supported the robustness and consistency of the observed inverse association between dietary Mn intake and T2D risk. The ability of the meta-regression model to explain nearly all between-study heterogeneity underscores the influence of study-level characteristics on effect estimates. Moreover, the narrow prediction interval suggests that future studies are likely to observe a similar protective trend, reinforcing the generalizability of the findings. The leave-one-out influence analysis further confirmed that no single study disproportionately influenced the results, strengthening confidence in the dose-response relationship.

### 4.4 Strengths and limitations

The strengths of this study mainly lie in its robust design and comprehensive approach to examining the link between dietary Mn intake and the risk of T2D and CVD. Leveraging data from the UK Biobank, one of the largest cohorts worldwide, ensures high data quality and statistical power. Additionally, our study benefits from the integration of both observational data and meta-analytic evidence, enabling a thorough assessment of Mn's role in chronic disease prevention. This dual approach enhances the interpretability and generalizability of findings and provides valuable insights for future research that could inform dietary guidelines.

However, there are limitations to consider. First, dietary intakes were assessed through online 24 -h dietary recalls, which may be susceptible to recall bias and day-to-day variations. To mitigate this issue, we computed the average intake from multiple instances of the Oxford WebQ when available, as this method has been shown to reduce random error and improve the reliability of usual intake estimates [33]. While single-day recalls may be less precise for micronutrients, validation studies have demonstrated that the Oxford WebQ performs with acceptable validity for key nutrients when benchmarked against objective biomarkers [33]. Furthermore, the reproducibility of dietary assessments in UK Biobank is widely consistent with prior prospective studies using conventional methods [34]. Despite these efforts, residual measurement error may persist and could attenuate true associations. Our sensitivity analyses did not indicate a substantial impact of intake variability on the observed estimates, but future studies should incorporate biomarker-based assessments of Mn status to enhance exposure precision.

Second, Mn intake was examined as an aggregate exposure, and we did not evaluate source-specific associations, which may have differential effects on T2D and CVD risk.

Third, although our UK Biobank analysis was supported by sensitivity checks and descriptive comparisons between included and excluded participants, it is characterized by significant exclusions and employed a complete-case approach, without the application of inverse probability weighting or multiple imputation. This approach may result in selection or collider bias associated with dietary recall participation and missing covariates. As such, residual bias cannot be entirely ruled out and should be considered when interpreting the findings.

Fourth, although we adjusted for wide range of confounders, including the overall diet quality via AHEI-2010, the possibility of residual confounding cannot be ruled out. Considering the potential for residual confounding by overall diet quality, future studies should prioritize rigorous adjustment using validated dietary indices to better isolate the independent effects of micronutrients like Mn. Lastly, the observational design of the current study limits causal inferences.

Last but not least, while the “some concern” risk of bias underscores the need for cautious interpretation, the significant reduction in residual heterogeneity and the narrow prediction interval suggest that the inverse association between dietary Mn intake and T2D risk is consistent across diverse cohorts, supporting the robustness and potential generalizability of the observed protective effect.

## 5 Conclusions

Our meta-analysis of >270,000 individuals from prospective cohorts but not our UK Biobank analysis, suggests that increasing dietary Mn intake above the level of adequate intake may be an effective strategy for T2D prevention. In contrast, the association between dietary Mn and CVD risk reduction appears to be modest.

## CRediT authorship contribution statement

**G.G.G**: Conceptualization, Methodology, formal analysis, literature screening, Writing- Original draft preparation. **B.Y.**: Conceptualization, visualization, Writing- Reviewing and Editing, formal analysis. **A.J.G.**: formal analysis, Writing- Reviewing and Editing. **A.Y.**: Formal analysis, Writing- Reviewing and Editing. **J.L.**: Formal analysis, Writing- Reviewing and Editing. **V.W.K.C.**: Methodology, visualization, literature screening. **M.S.W.**: Formal analysis, Writing- Reviewing and Editing. **S.L.**: Methodology, supervision, Writing- Reviewing and Editing. **K.K.H.L.**: Conceptualization, methodology, Writing- Reviewing and Editing, supervision.

## Ethical approval

The UK Biobank study was approved in 2011 by the North West Multi-centre Research Ethics Committee (MREC) (Reference number: 11/NW/0382) as a Research Tissue Bank (RTB) approval. All participants provided written informed consent.

## Declaration of Generative AI and AI-assisted technologies in the writing process

Not applicable.

## Funding

Dr Kenneth Lo was funded by Start-up Fund for New Recruits (Grant reference BE91). The funder had no role in study implementation and interpretation of findings.

## Declaration of competing interest

The authors declare that they have no competing interests.

## Footnotes

Appendix A Supplementary material related to this article can be found, in the online version, at doi:https://doi.org/10.1016/j.jnha.2025.100754.
