# Supplementary material for: Dietary manganese, type 2 diabetes, and cardiovascular disease: A UK Biobank cohort study and meta-analysis of over 270,000 individuals
Source: J Nutr Health Aging. 2025 Dec 10;30(2):100754. doi: 10.1016/j.jnha.2025.100754 (PMC12756635; doi:10.1016/j.jnha.2025.100754)
Supplement: Supplementary file 1 [file mmc1.docx]

**Supplementary material**

Contents

[Supplementary Methods 3](#_Toc215231096)

[Supplementary Table S1 Characteristics of included Vs. excluded participants with dietary assessments 6](#_Toc215231097)

[Supplementary Table S2 Quality assessment of included studies in meta-analysis using Newcastle-Ottawa Scale 9](#_Toc215231098)

[Supplementary Table S3 Risk of bias assessment of included studies in meta-analysis using ROBINS-E assessment tool 9](#_Toc215231099)

[Supplementary Table S4 GRADE certainty ratings for T2D and CVD outcomes from dose-response meta-analysis 10](#_Toc215231100)

[Supplementary Table S5 Interaction p-values and FDR-adjusted significance for Mn intake across key covariates and outcomes (T2D and Total CVD) 10](#_Toc215231101)

[Supplementary Table S6 Hazard ratios (HRs) and 95% confidence intervals (CIs) on the association of energy-adjusted dietary Mn intake with subtypes of total CVD & CVD mortality 11](#_Toc215231102)

[Supplementary Table S7 Sensitivity analysis of the association between original dietary Mn intake and T2D incidence 12](#_Toc215231103)

[Supplementary Table S8 Sensitivity analysis for the association of energy-adjusted dietary Mn intake with T2D incidence, total CVD, and CVD mortality in samples restricted to participants with specific number of dietary assessments 13](#_Toc215231104)

[Supplementary Table S9 Proportional hazard (PH) assumption tests using Schoenfeld residuals for T2D, CVD, and CVD mortality outcomes 14](#_Toc215231105)

[Supplementary Table S10 Hazard ratios (95% CI) of T2D, CVD, and CVD Mortality from lagged analysis by excluding first 2 years of follow‑up 15](#_Toc215231106)

[Supplementary Table S11 Competing risk regression of energy-adjusted Mn intake with CVD incidence, CVD mortality, and CVD subtypes 16](#_Toc215231107)

[Supplementary Table S12 Summary of non-linear analysis results for T2D, Total CVD incidence, and CVD Mortality: Risk Ratios, trend P-values, and E-values (Q5 vs Q1) 17](#_Toc215231108)

[Supplementary Table S13 Baseline characteristics of included studies in the dose-response meta-analysis of Mn intake and risk of T2D and total CVD 18](#_Toc215231109)

[Supplementary Table S14 Associations of dietary Mn intake with T2D and/or total CVD in included studies 19](#_Toc215231110)

[Supplementary Table S15 Meta-regression analysis to identify potential sources of heterogeneity in the association between Mn intake and T2D risk 20](#_Toc215231111)

[Supplementary Fig. S1 Participant selection Flowchart for UKB analysis 22](#_Toc215231112)

[Supplementary Fig. S2 Flow chart of study selection process for systematic review and meta-analysis 23](#_Toc215231113)

[Supplementary Fig. S3 Restricted cubic spline for dose-response meta-analysis of original dietary manganese intake and hazard ratio of T2D incidence for overall sample, men, and women. 24](#_Toc215231114)

[Supplementary Fig. S4 Leave-one-out sensitivity analysis plot for the dose-response meta-analysis on dietary manganese and T2D risk. 25](#_Toc215231115)

[Supplementary Fig. S5 Funnel plot with Egger’s regression line for publication bias in T2D meta-analysis 25](#_Toc215231116)

[Supplementary Fig. S6 Restricted cubic spline for dose-response meta-analysis of original dietary manganese intake and hazard ratio of total CVD for overall sample (a), men (b), and women (c). 26](#_Toc215231117)

[Supplementary References 27](#_Toc215231118)

# Supplementary Methods

**Measurement of dietary Mn:** Additional information on dietary assessment is provided here.

The Oxford WebQ is a quantitative dietary assessment tool that gathers information on 206 food items and 32 types of drinks consumed over the previous day and was validated for various nutrients against biomarkers [1] and showed acceptable reproducibility when compared to interviewer-based 24-hour recall questionnaires [2]. Participants were invited for dietary assessment at baseline and four follow-ups during the period of April 2009 and to June 2012. Collected dietary intake data were converted to nutrient and energy intake by the UK Biobank via built-in nutrient-conversion algorithms [3]. The manganese intake from supplements were not included in the calculation of diet-related nutrients. Thus, no supplement information was incorporated in the current analysis. Manganese was not one of the categories in the Vitamin and/or mineral supplement use variable. As a result, no participants were excluded based on supplement use.

**Other covariates**: Additional information on baseline covariates is included here.

Participants self-reported race/ethnicity was classified into five groups: White, Black, Asian, Mixed, and Other. Socioeconomic status (SES) was measured using Townsend deprivation index (TDI), a composite measure that included household income, employment status, and housing tenure. Educational attainment was measured by the highest level of education completed, categorized into groups ranging from no formal qualifications to higher education. Smoking status was determined through self-report, with participants categorized as current smokers, previous smokers, or never smoked. Similarly, alcohol consumption categorized into current drinkers, previous drinkers, and never-drinkers groups. Physical activity levels were measured using the International Physical Activity Questionnaire (IPAQ), where participants reported their activity over the past week, allowing for the computation of total physical activity in metabolic equivalent (MET) minutes per week [4]. Family history of T2D/CVD was assessed through self-reports regarding diagnoses in immediate family members. Hypertension status was defined by a systolic blood pressure (SBP) ≥140 mmHg, a diastolic blood pressure (DBP) ≥90 mmHg, or a self-reported history of hypertension, self-reported use of antihypertensive medications, or diagnoses at hospital based on ICD-10 codes I10. Body mass index (BMI) was calculated as the ratio of weight (kg) to height squared (m^2^), while waist circumference (WC) was measured in centimeters.

Diet quality was assessed using the Alternative Healthy Eating Index 2010 (AHEI-2010), derived from participants’ Oxford WebQ 24-hour dietary recalls in the UK Biobank. Nutrient intakes (Category 100117) and food-group consumption (Category 100118) were averaged across all available WebQs for each participant to estimate usual intake. We excluded implausible energy reporters and standardized all intakes to grams per day. Eleven AHEI components were computed: vegetables (excluding potatoes), fruit (excluding juice), whole grains, sugar-sweetened beverages (SSBs + fruit juice), nuts and legumes, red and processed meat, trans fat, long-chain n-3 (PUFA), total PUFA, sodium, and alcohol [5]. Food-group variables were constructed using specific UK Biobank data-fields—for example, vegetables from fields 26123, 26098, 26146, 26125, 26115, 26147, 26065, and 26143 (excluding 26118–26120 for potatoes); fruits from 26089–26094 (excluding 26095 for juice); whole-grain foods from 26074, 26114, 26076–26078, 26105 (with 26071 mixed bread counted as 50% whole-grain); nuts and legumes from 26101, 26106–26108; red and processed meat from 26066, 26100, 26117, and 26122; and oily fish from 26109 as a proxy for EPA + DHA. Trans fat (26155) and PUFA (26015 + 26016) were expressed as % of energy (26002). Sodium (26052) was ranked into deciles (lower = better). Alcohol (26030) was converted to drinks/day using 10–14 g ethanol per drink and scored using sex-specific J-shaped criteria. Each component was scored 0-10, linearly scaled between predefined minimum and maximum cut-points (e.g., 10 points for ≥ 5 servings/day vegetables, 0 for none). The total AHEI-2010 score, ranging 0-110, was computed as the sum of the 11 component scores, with higher values reflecting greater adherence to a healthy dietary pattern.

Dietary antioxidant intake was assessed using data from the Oxford WebQ 24-hour dietary recall questionnaires in the UK Biobank (Category 100117, “Estimated nutrients yesterday”). We constructed a Dietary Antioxidant Index (DAI) following the approach by Wright et al. (2004) [6]. For each participant, daily intakes of vitamin C (field 26023, mg/day), vitamin E (26028, mg/day), selenium (26058, µg/day), α-carotene (26038, µg/day), β-carotene (26039, µg/day), and total carotene (26027, µg/day) were extracted. When multiple WebQ assessments were available, nutrient intakes were averaged across all completed recalls to reduce within-person variability. Each nutrient was then adjusted for total energy intake (field 26002, kJ/day) using the residual method, with the residuals plus cohort mean values retained to represent energy-adjusted intakes. These adjusted values were standardized to z-scores within the analytic sample.

Because the three carotenoid variables were highly correlated, we used principal component analysis (PCA) to derive a single carotenoid component score (PC1), representing the first principal component of α-, β-, and total carotene. The final DAI was computed as the sum of standardized scores for PC1 (carotenoids), vitamin E, selenium, and vitamin C, each contributing equally to the index. The DAI was subsequently adjusted for total energy intake using the residual method and re-standardized (mean = 0, SD = 1) to facilitate interpretation. Higher DAI values indicate a diet richer in antioxidant nutrients.

**Outcome Ascertainment**: Detailed information on outcome ascertainment is included here.

T2D incidence was defined based on the National Diabetes Data Group classification or ICD10-E11 or previous physician diagnosis of diabetes. T2D incidence was defined as a fasting plasma glucose level of at least 7.0 mmol/L (no caloric intake for at least 8 h), the presence of diabetes symptoms and a random plasma glucose level of at least 11.1 mmol/L, or a 2-hour plasma glucose level of at least 11.1 mmol/L during an oral glucose tolerance test (administered with 75 g of anhydrous glucose dissolved in water, as described by WHO). An HbA1c level of at least 6.5 % was also used as an optional diagnostic criterion. This criteria has been used by other published UKB studies [7]. Total CVD (incidence and/or mortality) was identified through linkage to Hospital Episode Statistics for England, Scottish Morbidity Record data for Scotland, and the Patient Episode Database for Wales, a validated method for CVD ascertainment [8]. Total CVD consisted of coronary heart disease (CHD) recorded using International Classification of Diseases-10 (ICD-10 codes I20–I25; stroke (I60, I61, I63, I64), and myocardial infarction (I21, I22, I23, I241, I252). We estimated participant follow-up time from baseline visit until age at last follow-up, first diagnosis of T2D/CVD outcome, loss to follow-up or death (applicable to CVD mortality only), or the census date (2022-12-17), whichever was the earliest.

**Statistical analysis**

In the T2D analytic cohort, Model 1 was adjusted for age, sex (only overall sample) and race/ethnicity. Model 2 was further adjusted for dietary energy intake. Model 3 was further adjusted for socioeconomic status, education, smoking status, alcohol drinking, physical activity measured by IPAQ, family history of T2D, hypertension at baseline, CVD at baseline, and BMI. For the CVD analytic cohort, family history of T2D and CVD at baseline were replaced with family history of CVD and T2D at baseline, respectively. In the same manner as the main analysis, sex-specific analysis was conducted in both analytic cohorts.

Sensitivity analyses of the associations between original (non-energy-adjusted) dietary Mn and T2D risk, CVD risk, and CVD mortality risk were conducted to compare with associations based on the energy-adjusted Mn intake. Based on the procedures by Desquilbet and Mariotti [9], we also fitted restricted cubic splines (RCS) with five knots at 5%, 25%, 50%, 75%, and 95% to model non-linear dose-response associations between Mn intake and risk of T2D, total CVD and CVD mortality.

**Meta-analysis of prospective cohorts**

**Search strategy**

The keywords used in the literature search were: “manganese” AND (“Type 2 diabetes” OR “diabetes” OR “cardiovascular disease” OR “coronary heart disease” OR “myocardial infarction” OR “heart failure” OR “stroke” OR “cerebrovascular disease” OR “ischemic heart disease” OR “mortality” OR “death”) AND (“cohort” OR “prospective” OR “follow-up” OR “longitudinal” OR “population”). Our search was restricted to studies conducted in humans, and no restriction was imposed with respect to the language of the publications. Retrieved relevant articles were further reviewed for their references to identify and include additional studies.

**Study Selection and data extraction**

We excluded reviews, non-cohort observational studies, intervention studies, protocols, conference abstracts, and editorials. To select studies that meet the inclusion criteria, we used a two-step selection process. First, two independent investigators (G.G.G. and V.C.) conducted title and abstract screening of retrieved studies. Then, potentially relevant articles went through full text evaluation.

Data were extracted using a standardized form by two independent investigators (G.G.G. and V.C.). Any discrepancies during data extraction were agreed through group discussion with a third investigator (K.L.).

**Dose-response meta-analysis**

To estimate the pooled exposure-outcome curve from the extracted dose-response data, a two-stage random effects meta-analysis was conducted. The first stage involved determining the dose-response association between the adjusted log relative risks and the levels of the exposure for each included study. In the second stage of analysis, study-specific estimates were pooled using established methods for multivariate meta-analysis. To examine potential nonlinear associations, we used restricted cubic splines with 3 knots at fixed percentiles 25%, 50% and 75% of the distribution. Our non-linear analysis enabled us to determine any threshold or plateau levels of Mn intake beyond which Mn intake might not exert any effect on T2D/CVD risk. We also did subgroup analyses to see whether the statistical relationship observed in the overall sample stands in subgroups of the sample.

Moreover, a series of sensitivity analysis including leave-one-out analysis, meta-regression, and prediction intervals was conducted to assess robustness of the dose-response meta-analysis. To further assess the influence of individual studies, we conducted a leave-one-out analysis, sequentially removing each study and recalculating the pooled effect. The meta-regression was conducted using study-level moderators including dietary assessment method, geographic region, sex, and follow-up duration. A prediction interval was calculated from the overall meta-analysis model to assess the expected range of effects in future studies

# Supplementary Table S1 Characteristics of included Vs. excluded participants with dietary assessments

| **Variable** | **Excluded** | **Included** | **P-test** |
| --- | --- | --- | --- |
| n | 45756 | 165194 |  |
| Age, mean (SD) | 57.22 (7.79) | 55.76 (7.96) | <0.001 |
| Sex, % |  |  | <0.001 |
| Male | 26735 (58.4) | 89469 (54.2) |  |
| Female | 19021 (41.6) | 75725 (45.8) |  |
| Ethnicity, % |  |  | <0.001 |
| Asian | 982 (2.2) | 2547 (1.5) |  |
| Black | 790 (1.7) | 1820 (1.1) |  |
| Mixed | 301 (0.7) | 937 (0.6) |  |
| Other | 647 (1.4) | 1580 (1.0) |  |
| White | 42756 (94.0) | 158310 (95.8) |  |
| BMI, mean (SD) | 28.02 (5.30) | 26.69 (4.42) | <0.001 |
| SES, mean (SD) | -1.35 (2.98) | -1.63 (2.85) | <0.001 |
| Educational status, % |  |  | <0.001 |
| Higher education | 15410 (33.7) | 74314 (45.0) |  |
| Lower education | 30262 (66.3) | 90880 (55.0) |  |
| Smoking, % |  |  | <0.001 |
| Current | 3909 (8.5) | 12637 (7.6) |  |
| Never | 25040 (54.7) | 94508 (57.2) |  |
| Previous | 16807 (36.7) | 58049 (35.1) |  |
| Alcohol drinking, % |  |  | <0.001 |
| Current | 41757 (91.3) | 155657 (94.2) |  |
| Never | 2149 (4.7) | 4929 (3.0) |  |
| Previous | 1850 (4.0) | 4608 (2.8) |  |
| IPAQ, mean (SD) | 1.10 (0.76) | 1.22 (0.73) | <0.001 |
| Family history of diabetes, % |  |  | <0.001 |
| No | 27729 (75.5) | 118126 (82.1) |  |
| Yes | 8992 (24.5) | 25834 (17.9) |  |
| Multivitamin, % |  |  | <0.001 |
| No | 23653 (51.7) | 83483 (50.5) |  |
| Yes | 22103 (48.3) | 81711 (49.5) |  |
| Energy, mean (SD) | 2033.64 (697.51) | 2074.07 (579.39) | <0.001 |
| Mn, mean (SD) | 4.12 (1.64) | 4.25 (1.51) | <0.001 |

# Supplementary Table S2 Quality assessment of included studies in meta-analysis using Newcastle-Ottawa Scale

| **Author, year** | **Selection** | | | | **Comparability** | **Outcome** | | | **Overall quality** |
| --- | --- | --- | --- | --- | --- | --- | --- | --- | --- |
|  | **Representativeness of the exposed cohort** | **Selection of the non-exposed cohort** | **Ascertainment of exposure** | **Demonstration that outcome of interest was not present at start of study** | **Comparability of cohorts on the basis of the design or analysis controlled for confounders** | **Assessment of outcome** | **Was follow-up long enough for outcomes to occur** | **Adequacy of follow-up of cohorts** |  |
| Du et al., 2018 | * | * |  | * | ** |  | * | * | Good |
| Eshak et al., 2021 | * | * | * | * | ** | * |  | * | Good |
| Gong et al., 2020 |  | * | * | * | ** | * | * | * | Good |
| Meishuo et al., 2022 | * | * |  | * | ** | * | * |  | Good |
| Yazdanpanah et al., 2024 |  | * |  | * | ** | * | * |  | Fair |

# Supplementary Table S3 Risk of bias assessment of included studies in meta-analysis using ROBINS-E assessment tool

| **Study** | **Confounding** | **Selection of participants** | **Classification of exposure** | **Departure from exposure** | **Missing data** | **Outcome measurement** | **Reporting bias** | **Overall risk** |
| --- | --- | --- | --- | --- | --- | --- | --- | --- |
| Du et al., 2018 | Some concerns | Low | Some concerns | Low | Low | Low | Low | Some concerns |
| Eshak et al., 2021 | Some concerns | Low | Some concerns | Low | Low | Some concerns | Low | Some concerns |
| Gong et al., 2020 | Some concerns | Low | Some concerns | Low | Low | Some concerns | Low | Some concerns |
| Meishuo et al., 2022 | Some concerns | Low | Some concerns | Low | Low | Low | Low | Some concerns |
| Yazdanpanah et al., 2024 | Some concerns | Low | Some concerns | Some concerns | Low | Low | Low | Some concerns |

| Outcome | Risk of bias | Inconsistency | Indirectness | Imprecision | Publication bias | Overall certainty |
| --- | --- | --- | --- | --- | --- | --- |
| T2D | Moderate | Moderate (I² = 57.8%) | Low | Low | Undetected | Low |
| CVD | Moderate | Low (I² = 0.0%) | Low | Moderate (borderline CI, non-significant result) | Undetected | Low |

# Supplementary Table S4 GRADE certainty ratings for T2D and CVD outcomes from dose-response meta-analysis

# Supplementary Table S5 Interaction p-values and false discovery rate (FDR)-adjusted significance for Mn intake across key covariates and outcomes (T2D and Total CVD)

| **T2D** | | |
| --- | --- | --- |
| **Interaction term (Variable X Mn)** | **Raw p-value** | **FDR-adjusted p-value** |
| Baseline CVD | 0.18 | 0.31 |
| BMI | 0.003 | 0.01 |
| Sex | 0.66 | 0.66 |
| Age | 0.23 | 0.31 |
| **Total CVD** | | |
| Interaction term (X Mn) | Raw p-value | FDR-adjusted p-value |
| Baseline diabetes | 0.86 | 0.86 |
| BMI | 0.43 | 0.58 |
| Sex | 0.35 | 0.55 |
| Age | 0.03 | 0.24 |

#

# Supplementary Table S6 Hazard ratios (HRs) and 95% confidence intervals (CIs) on the association of energy-adjusted dietary Mn intake with subtypes of total CVD & CVD mortality

| **HR** | **Q1** | **Q2** | **Q3** | **Q4** | **Q5** | **Continuous** | **P for trend** |  |
| --- | --- | --- | --- | --- | --- | --- | --- | --- |
| Incident CVD | | | | | | | | |
| MI (N=2,882) | ref | 0.91 (0.81, 1.02) | 0.83 (0.73, 0.93) | 1.01 (0.89, 1.14) | 0.96 (0.84, 1.09) | 1 (0.97, 1.04) | 0.96 |  |
| CHD (N=8,536) | ref | 0.95 (0.89, 1.01) | 0.92 (0.86, 0.98) | 1.01 (0.95, 1.08) | 0.98 (0.91, 1.05) | 1 (0.99, 1.02) | 0.65 |  |
| HF (N=3,194) | ref | 0.96 (0.86, 1.08) | 0.96 (0.86, 1.07) | 0.98 (0.87, 1.11) | 0.97 (0.85, 1.1) | 0.99 (0.96, 1.02) | 0.64 |  |
| Stroke (N=588) | ref | 0.9 (0.69, 1.18) | 0.99 (0.76, 1.29) | 1.06 (0.81, 1.4) | 0.92 (0.69, 1.24) | 1 (0.92, 1.07) | 0.90 |  |
| CVD mortality | | | | | | | | |
| MI (N=273) | ref | 0.56 (0.39, 0.81) | 0.74 (0.52, 1.05) | 0.72 (0.49, 1.04) | 0.71 (0.48, 1.04) | 0.93 (0.84, 1.03) | 0.17 |  |
| CHD (N=158) | ref | 0.52 (0.38, 0.72) | 0.89 (0.67, 1.19) | 0.82 (0.61, 1.11) | 0.84 (0.61, 1.15) | 0.98 (0.9, 1.07) | 0.67 |  |
| HF (N=27) | ref | 0.47 (0.27, 0.8) | 1.03 (0.66, 1.61) | 0.74 (0.45, 1.23) | 0.9 (0.54, 1.49) | 1 (0.87, 1.15) | 0.98 |  |
| Stroke (N=84) | ref | 0.85 (0.43, 1.67) | 1.04 (0.54, 2.03) | 1.25 (0.64, 2.45) | 0.92 (0.43, 1.99) | 0.99 (0.81, 1.21) | 0.95 |  |

Adjusted for age, sex, race/ethnicity, dietary energy, AHEI-2010, DAI, socioeconomic status, education, smoking status, alcohol drinking, physical activity measured by IPAQ, family CVD history, hypertension at baseline, diabetes at baseline, and body mass index (BMI). Abbreviations: AHEI-2010; Alternative Healthy Eating Index-2010, DAI; Dietary antioxidant intake, CI; Confidence interval, CHD: Coronary heart disease, CVD; Cardiovascular disease, HF: Heart failure, HR; Hazard ratio, MI: Myocardial infraction, ref; reference.

# Supplementary Table S7 Sensitivity analysis of the association between original dietary Mn intake and T2D incidence

| **HR** | **Q1** | **Q2** | **Q3** | **Q4** | **Q5** | **Continuous** | **P for trend** |
| --- | --- | --- | --- | --- | --- | --- | --- |
| T2D | ref | 0.97 (0.88, 1.06) | 0.97 (0.88, 1.07) | 0.88 (0.79, 0.98) | 0.93 (0.82, 1.05) | 0.98 (0.95, 1.00) | 0.07 |
| Total CVD | ref | 0.95 (0.89,1.01) | 0.93 (0.88,0.99) | 0.98 (0.92,1.04) | 0.99 (0.92,1.06) | 1.0 (0.99,1.02) | 0.61 |
| CVD mortality | ref | 0.96 (0.74,1.26) | 0.90 (0.68,1.20) | 0.75 (0.55,1.03) | 0.91 (0.65,1.28) | 0.98 (0.91,1.06) | 0.66 |

Adjusted for age, sex, race/ethnicity, dietary energy, socioeconomic status, education, smoking status, alcohol drinking, physical activity measured by IPAQ, family history of diabetes (family history of CVD for CVD outcomes), hypertension at baseline, diabetes at baseline (CVD at baseline for CVD outcomes), AHEI-2010, DAI, and body mass index (BMI). **Abbreviations**: AHEI-2010; Alternative Healthy Eating Index-2010, CI; Confidence interval, CVD; Cardiovascular disease, DAI; Dietary antioxidant intake, HR; Hazard ratio, Q; quintile, ref; reference

# Supplementary Table S8 Sensitivity analysis for the association of energy-adjusted dietary Mn intake with T2D incidence, total CVD, and CVD mortality in samples restricted to participants with specific number of dietary assessments

| **Sample restricted to number of dietary assessments** | **HR (95% CIs)** | | | | | | |
| --- | --- | --- | --- | --- | --- | --- | --- |
|  | **Q1** | **Q2** | **Q3** | **Q4** | **Q5** | **Continuous** | **P-value** |
| **T2D** | | | | | | | |
| ≥2 (N=101,390) | ref | 0.99 (0.88, 1.12) | 0.95 (0.84, 1.08) | 0.92 (0.80, 1.05) | 0.95 (0.82, 1.10) | 0.97 (0.93, 1.01) | 0.193 |
| ≥3 (N=63,300) | ref | 1.00 (0.86, 1.16) | 0.93 (0.79, 1.10) | 0.86 (0.72, 1.03) | 0.87 (0.72, 1.06) | 0.96 (0.91, 1.02) | 0.152 |
| ≥4 (N=29,220) | ref | 1.07 (0.85, 1.34) | 1.04 (0.81, 1.33) | 0.91 (0.69, 1.19) | 0.85 (0.63, 1.16) | 0.94 (0.86, 1.02) | 0.154 |
| ≥5 (N=4,620) | ref | 1.05 (0.60, 1.86) | 0.80 (0.41, 1.53) | 1.09 (0.57, 2.06) | 0.81 (0.37, 1.76) | 0.87 (0.69, 1.10) | 0.236 |
| **Total CVD** | | | | | | | |
| ≥2 (N=100,581) | ref | 0.9 (0.83, 0.97) | 0.93 (0.86, 1.01) | 1.03 (0.95, 1.12) | 0.98 (0.9, 1.07) | 1.01 (0.98, 1.03) | 0.648 |
| ≥3 (N=62,752) | ref | 0.9 (0.82, 0.99) | 0.97 (0.87, 1.07) | 1.07 (0.97, 1.19) | 1.02 (0.91, 1.14) | 1.02 (0.99, 1.06) | 0.151 |
| ≥4 (N=28,978) | ref | 0.93 (0.8, 1.09) | 1 (0.85, 1.17) | 1.21 (1.03, 1.41) | 1.14 (0.96, 1.35) | 1.06 (1.01, 1.11) | 0.027 |
| ≥5 (N=4,594) | ref | 0.89 (0.61, 1.29) | 1.01 (0.69, 1.46) | 0.94 (0.63, 1.41) | 0.94 (0.61, 1.44) | 0.97 (0.85, 1.1) | 0.615 |
| **CVD Mortality** | | | | | | | |
| ≥2 (N=100,581) | ref | 0.59 (0.4, 0.86) | 0.88 (0.62, 1.25) | 0.66 (0.44, 0.98) | 0.77 (0.51, 1.15) | 0.95 (0.84, 1.07) | 0.391 |
| ≥3 (N=62,752) | ref | 0.54 (0.33, 0.87) | 0.89 (0.58, 1.38) | 0.63 (0.38, 1.04) | 0.58 (0.33, 1) | 0.83 (0.7, 0.97) | 0.023 |
| ≥4 (N=28,978) | ref | 0.83 (0.41, 1.68) | 1.18 (0.6, 2.33) | 1.12 (0.54, 2.31) | 0.65 (0.26, 1.61) | 0.86 (0.66, 1.1) | 0.232 |
| ≥5 (N=4,594) | ref | 1.22 (0.24, 6.13) | 1.09 (0.21, 5.79) | 1.31 (0.23, 7.57) | 0.52 (0.04, 6.18) | 0.68 (0.35, 1.34) | 0.264 |

Adjusted for age, sex, race/ethnicity, dietary energy, socioeconomic status, education, smoking status, alcohol drinking, physical activity measured by IPAQ, family CVD history (for CVD outcomes only), hypertension at baseline, diabetes at baseline (for CVD outcomes only), family diabetes history (for T2D outcome only), CVD at baseline (for T2D outcome only), AHEI-2010, DAI, and body mass index (BMI). **Abbreviations**: AHEI-2010; Alternative Healthy Eating Index-2010, CI; Confidence interval, CVD; Cardiovascular disease, DAI; Dietary antioxidant intake Q; quintile, ref; reference group

# Supplementary Table S9 Proportional hazard (PH) assumption tests using Schoenfeld residuals for T2D, CVD, and CVD mortality outcomes

| **Variable** | **P-value** | | |
| --- | --- | --- | --- |
|  | **T2D incidence** | **CVD incidence** | **CVD mortality** |
| Energy adjusted Mn intake (quintile) | 0.79 | 0.92 | 0.57 |
| Age | 0.52 | 0.44 | 0.10 |
| Sex | 0.85 | 0.003 | 0.10 |
| Ethnicity | 0.07 | 0.51 | 0.87 |
| Energy intake | 0.87 | 0.56 | 0.75 |
| AHEI-2010 | 0.78 | 0.15 | 0.85 |
| Dietary antioxidant intake (q5 ) | 0.66 | 0.003 | 0.42 |
| Socioeconomic status | 0.04 | 0.09 | 0.03 |
| Education | 0.46 | 0.04 | 0.61 |
| Smoking | 0.02 | 0.76 | 0.63 |
| Alcohol drinking | 0.67 | 0.93 | 0.98 |
| IPAQ | 0.01 | 0.001 | 0.07 |
| Family history | 0.10 | 0.05 | 0.54 |
| Baseline HTN | 0.10 | 0.51 | 0.79 |
| Baseline CVD (diabetes for CVD analysis sample) | 0.68 | 0.82 | 0.14 |
| BMI | 0.004 | 0.21 | 0.43 |
| Multivitamin | 0.29 | - | - |
| GLOBAL | 0.10 | 0.004 | 0.15 |

# Supplementary Table S10 Hazard ratios (95% CI) of T2D, CVD, and CVD Mortality from lagged analysis by excluding first 2 years of follow‑up

| **Model** | **Q1** | **Q2** | **Q3** | **Q4** | **Q5** | **Continuous** | **P-value** |
| --- | --- | --- | --- | --- | --- | --- | --- |
| **T2D incidence** | | | | | | | |
| Model 1 | ref | 0.75 (0.69, 0.81) | 0.66 (0.61, 0.72) | 0.60 (0.55, 0.66) | 0.55 (0.50, 0.60) | 0.84 (0.82, 0.86) | <0.001 |
| Model 2 | ref | 0.83 (0.77, 0.90) | 0.78 (0.72, 0.85) | 0.74 (0.68, 0.81) | 0.70 (0.64, 0.78) | 0.91 (0.88, 0.93) | <0.001 |
| Model 3 | ref | 0.93 (0.85, 1.01) | 0.91 (0.83, 1.00) | 0.92 (0.83, 1.01) | 0.91 (0.82, 1.01) | 0.98 (0.95, 1.00) | 0.07 |
| **CVD incidence** | | | | | | | |
| Model 1 | ref | 0.87 (0.82, 0.92) | 0.81 (0.77, 0.86) | 0.84 (0.79, 0.89) | 0.78 (0.74, 0.83) | 0.94 (0.93, 0.95) | <0.001 |
| Model 2 | ref | 0.91 (0.86, 0.96) | 0.87 (0.82, 0.92) | 0.92 (0.87, 0.98) | 0.88 (0.83, 0.94) | 0.97 (0.96, 0.99) | <0.01 |
| Model 3 | ref | 0.95 (0.9, 1.01) | 0.94 (0.88, 0.99) | 1.02 (0.96, 1.08) | 0.99 (0.92, 1.05) | 1 (0.99, 1.02) | 0.60 |
| **CVD Mortality** | | | | | | | |
| Model 1 | ref | 0.49 (0.37, 0.65) | 0.7 (0.55, 0.9) | 0.64 (0.5, 0.82) | 0.64 (0.5, 0.82) | 0.9 (0.84, 0.97) | 0.004 |
| Model 2 | ref | 0.51 (0.39, 0.68) | 0.75 (0.58, 0.97) | 0.7 (0.53, 0.92) | 0.73 (0.55, 0.97) | 0.94 (0.87, 1.02) | 0.121 |
| Model 3 | ref | 0.55 (0.42, 0.74) | 0.83 (0.64, 1.08) | 0.81 (0.61, 1.07) | 0.85 (0.64, 1.13) | 0.98 (0.91, 1.06) | 0.663 |

Model 1 was adjusted for age, sex, and race/ethnicity; Model 2 was further adjusted for dietary energy, AHEI-2020, and DAI; Model 3 was further adjusted for socioeconomic status, education, smoking status, alcohol drinking, physical activity measured by IPAQ, family history of CVD (for CVD outcomes only), hypertension at baseline, diabetes at baseline (for CVD outcomes only), family history of diabetes (for T2D outcome only), CVD at baseline (for T2D outcome only), and body mass index (BMI). **Abbreviations**: AHEI-2010; Alternative Healthy Eating Index-2010, CI; Confidence interval, CVD; Cardiovascular disease, DAI; Dietary antioxidant intake Q; quintile, ref; reference group

# Supplementary Table S11 Competing risk regression of energy-adjusted Mn intake with CVD incidence, CVD mortality, and CVD subtypes

| **Mn intake** | **CVD Incidence HR (95% CI), p** | **CVD Mortality HR (95% CI), p** | **MI HR (95% CI), p** | **CHD HR (95% CI), p** | **HF HR (95% CI), p** | **Stroke HR (95% CI), p** |
| --- | --- | --- | --- | --- | --- | --- |
| Q1 (Ref) | 1.00, - | 1.00, - | 1.00, - | 1.00, - | 1.00, - | 1.00, - |
| Q2 | 0.96 (0.91, 1.02), 0.16 | 0.56 (0.42, 0.74), <0.001 | 0.91 (0.82, 1.02), 0.12 | 0.96 (0.90, 1.02), 0.15 | 0.97 (0.87, 1.09), 0.61 | 0.91 (0.70, 1.19), 0.49 |
| Q3 | 0.95 (0.89, 1.00), 0.07 | 0.85 (0.65, 1.10), 0.21 | 0.83 (0.74, 0.94), <0.01 | 0.93 (0.87, 0.99), 0.03 | 0.97 (0.87, 1.09), 0.65 | 1.00 (0.76, 1.31), 1.00 |
| Q4 | 1.03 (0.97, 1.09), 0.43 | 0.83 (0.62, 1.10), 0.20 | 1.02 (0.90, 1.15), 0.79 | 1.02 (0.96, 1.09), 0.49 | 0.99 (0.88, 1.12), 0.92 | 1.07 (0.82, 1.41), 0.61 |
| Q5 | 1.00 (0.94, 1.07), 0.98 | 0.88 (0.66, 1.17), 0.37 | 0.97 (0.85, 1.11), 0.65 | 1.00 (0.93, 1.07), 0.91 | 0.99 (0.87, 1.12), 0.87 | 0.94 (0.71, 1.26), 0.69 |
| Continuous | 1.01 (0.99, 1.03), 0.31 | 0.99 (0.91, 1.08), 0.87 | 1.01 (0.97, 1.04), 0.76 | 1.01 (0.99, 1.03), 0.35 | 1.00 (0.97, 1.03), 0.95 | 1.00 (0.93, 1.08), 0.98 |

Hazard ratios (HR) and 95% confidence intervals (CI) are from Fine-gray competing risks regression models, adjusted for age, sex, ethnicity, energy intake, AHEI-2010, DAI, SES, education, smoking, drinking, physical activity (IPAQ), family history, baseline hypertension, BMI, and multivitamin use. Abbreviations: AHEI-2010; Alternative Healthy Eating Index-2010, CHD; Coronary heart disease, CI; Confidence interval, CVD; Cardiovascular disease, DAI; Dietary antioxidant intake, HF; Heart failure, HR; Hazard ratio, MI: Myocardial infraction, ref; reference.

# Supplementary Table S12 Summary of non-linear analysis results for T2D, Total CVD incidence, and CVD Mortality: Risk Ratios, trend P-values, and E-values (Q5 vs Q1)

| **Outcome** | **PLIN** | **PNL** | **RR (point) (95% CI)** | **E-value (point)** | **E-value (CI limit)** |
| --- | --- | --- | --- | --- | --- |
| T2D | 0.07 | 0.03 | 0.91 (0.82, 1.01) | 1.44 | 1.00 |
| CVD incidence | 0.61 | 0.01 | 0.99 (0.92, 1.05) | 1.13 | 1.00 |
| CVD mortality | 0.66 | 0.01 | 0.85 (0.64, 1.13) | 1.64 | 1.00 |

Abbreviations; CI, confidence interval; CVD, cardiovascular disease; E-value, minimum strength of association required for an unmeasured confounder to fully explain away the observed association; PNL, p-value for non-linear trend; PLIN, p-value for linear trend; RR, relative risk; T2D, type 2 diabetes

# Supplementary Table S13 Baseline characteristics of included studies in the dose-response meta-analysis of Mn intake and risk of T2D and total CVD

| **Author, year** | **Country** | **Cohort name** | **Sex** | **Age at entry** | **Follow up years** | **Sample size** | **Exposure; assessment method** | **Outcome** |
| --- | --- | --- | --- | --- | --- | --- | --- | --- |
| Du eta al., 2018 | China | Harbin People’s Health study (HPHS) and Harbin  Cohort Study on Diet, Nutrition and Chronic Noncommunicable  Diseases (HDNNCDS) | Both | 20-74 | 4.2 (HPHS), 5.3 (HDNNCDS) | 10,483 | Dietary manganese; Food frequency questionnaire | Type 2 diabetes incidence |
| Eshak et al., 2021 | Japan | Japan Collaborative Cohort Study (JACC) | Both | 40-79 | 5 | 19,862 | Dietary manganese; Food frequency questionnaire | Type 2 diabetes incidence |
| Gong et al., 2020 | USA | Women’s Health Initiative Observational Study (WHI-OS) | Women | 50-59 | 10.8 | 84,285 | Dietary manganese; Food frequency questionnaire | Type 2 diabetes incidence |
| Meishuo et al., 2022 | Japan | Japan Collaborative Cohort Study (JACC) | Both | 40-79 | 16.5 | 58,872 | Dietary manganese; Food frequency questionnaire | CVD mortality |
| Yazdanpanah et al., 2024 | Iran | Golestan Cohort Study | Both | 45-75 | 14.3 | 61,863 | Dietary intake; Food frequency questionnaire | CVD mortality |
| Gebretsadkan et al., 2025 (the current analysis) | UK | UK Biobank Study | Both | 40-69 | 10.8 (T2D), 10.6 (CVD) | 165,194 (T2D), 164,111 (CVD) | Dietary manganese intake: 24-hour recall | Type 2 diabetes incidence, total CVD, CVD mortality |

# Supplementary Table S14 Associations of dietary Mn intake with T2D and/or total CVD in included studies

|  |  |  |  | **Outcome** | | |
| --- | --- | --- | --- | --- | --- | --- |
| **Author, year** | **Study period** | **Sex** | **Mn intake** | **RR (95% CI)** | **Reference category** | **Measure of effect estimate** |
| Du eta al., 2018 | 2008-2012 (HPHS) | Both | 4.22-4.91 mg/day | 1.16 (0.81, 1.67) | <4.22 mg/day | Relative Risk |
|  |  |  | ≥4.91 mg/day | 0.52 (0.33, 0.82) |  |  |
|  | 2010-2015/16 (HDNNCDS) | Both | 4.27-4.9 8 mg/day | 0.99 (0.75, 1.31) | <4.27 mg/day |  |
|  |  |  | ≥4.98 mg/day | 0.61 (0.43, 0.88) |  |  |
| Eshak et al., 2021 | 1988/90 – 1993/95 | Men | Q2 (4.6 ± 0.5 mg/day) | 0.97 (0.65, 1.43) | Q1 (2.7 ± 0.7 mg/day) | Odds Ratio |
|  |  |  | Q3 (6.4 ± 0.6 mg/day) | 1.04 (0.67, 1.61) |  |  |
|  |  |  | Q4 (9.7 ± 1.8 mg/day) | 1.10 (0.64, 1.92) |  |  |
|  |  | Women | Q2 (4.1 ± 0.5 mg/day) | 0.74 (0.51, 1.06) | Q1 (2.5 ± 0.5 mg/day) |  |
|  |  |  | Q3 (5.9 ± 0.5 mg/day) | 0.62 (0.41, 0.94) |  |  |
|  |  |  | Q4 (8.6 ± 1.6 mg/day) | 0.53 (0.31, 0.88) |  |  |
| Gong et al., 2020 | 1993-2010 | Women | Q2 (2.49 ± 0.82 mg/day) | 0.93 (0.87, 1.00) | Q1 (2.17 ± 0.93 mg/day) | Hazard Ratio |
|  |  |  | Q3 (2.99 ± 0.78 mg/day) | 0.84 (0.78, 0.90) |  |  |
|  |  |  | Q4 (3.64 ± 0.78 mg/day) | 0.73 (0.68, 0.79) |  |  |
|  |  |  | Q5 (5.11 ± 1.25 mg/day) | 0.70 (0.65, 0.76) |  |  |
| Meishuo1 et al., 2022 | 1988/90-2009 | Men | Q2 (4.2 ± 0.5 mg/day) | 0.88 (0.75, 1.03) | Q1 (3.0 ± 0.4 mg/day) | Hazard Ratio |
|  |  |  | Q3 (5.6 ± 0.4 mg/day) | 1.04 (0.88, 1.23) |  |  |
|  |  |  | Q4 (7.1 ± 0.4 mg/day) | 0.86 (0.73, 1.05) |  |  |
|  |  |  | Q5 (10.0 ± 1.7 mg/day) | 0.89 (0.76, 1.05) |  |  |
|  |  | Women | Q2 (3.8 ± 0.5 mg/day) | 0.98 (0.84, 1.15) | Q1 (2.7 ± 0.3 mg/day) |  |
|  |  |  | Q3 (5.2 ± 0.3 mg/day) | 0.96 (0.81, 1.13) |  |  |
|  |  |  | Q4 (6.6 ± 0.4 mg/day) | 0.89 (0.75, 1.05) |  |  |
|  |  |  | Q5 (9.2 ± 1.6 mg/day) | 0.79 (0.67, 0.94) |  |  |
| Yazdanpanah et al., 2024 | 2004-2021 | Both | Q2 (3.79 ± 0.14 mg/1000 Kcal/day) | 0.92 (0.81, 1.04) | Q1 (2.96 ± 0.47 mg/1000 Kcal/day) | Hazard Ratio |
|  |  |  | Q3 (4.26 ± 0.12 mg/1000 Kcal/day) | 0.99 (0.87, 1.12) |  |  |
|  |  |  | Q4 (4.75 ± 0.16 mg/1000 Kcal/day) | 0.90 (0.79, 1.02) |  |  |
|  |  |  | Q5 (5.75 ± 0.77 mg/1000 Kcal/day) | 1.07 (0.87, 1.20) |  |  |
| Gebretsadkan et al., 2025 (T2D) |  | Men | Q2 (3.40 ± 0.22 mg/day) | 0.87 (0.78, 0.97) | Q1 (2.34 ± 0.51 mg/day) | Hazard Ratio |
|  |  |  | Q3 (4.12 ± 0.20 mg/day) | 0.85 (0.76, 0.95) |  |  |
|  |  |  | Q4 (4.90 ± 0.26 mg/day) | 0.80 (0.71, 0.90) |  |  |
|  |  |  | Q5 (6.59 ± 1.15 mg/day) | 0.79 (0.69, 0.90) |  |  |
|  |  | Women | Q2 (3.39 ± 0.22 mg/day) | 0.95 (0.84, 1.09) | Q1 (2.36 ± 0.50 mg/day) |  |
|  |  |  | Q3 (4.12 ± 0.20 mg/day) | 0.95 (0.83, 1.09) |  |  |
|  |  |  | Q4 (4.89 ± 0.26 mg/day) | 0.83 (0.71, 0.96) |  |  |
|  |  |  | Q5 (6.39 ± 1.00 mg/day) | 0.86 (0.73, 1.01) |  |  |
| Gebretsadkan et al., 2025 (CVD) |  | Men | Q2 (3.40 ± 0.22 mg/day) | 0.90 (0.83, 0.96) | Q1 (2.34 ± 0.51 mg/day) | Hazard Ratio |
|  |  |  | Q3 (4.12 ± 0.20 mg/day) | 0.88 (0.82, 0.95) |  |  |
|  |  |  | Q4 (4.90 ± 0.26 mg/day) | 0.89 (0.83, 0.96) |  |  |
|  |  |  | Q5 (6.59 ± 1.16 mg/day) | 0.89 (0.82, 0.97) |  |  |
|  |  | Women | Q2 (3.39 ± 0.22 mg/day) | 0.96 (0.88, 1.06) | Q1 (2.36 ± 0.50 mg/day) |  |
|  |  |  | Q3 (4.12 ± 0.20 mg/day) | 0.95 (0.86, 1.05) |  |  |
|  |  |  | Q4 (4.89 ± 0.26 mg/day) | 1.06 (0.95, 1.17) |  |  |
|  |  |  | Q5 (6.39 ± 1.00 mg/day) | 0.99 (0.89, 1.11) |  |  |

# Supplementary Table S15 Meta-regression analysis to identify potential sources of heterogeneity in the association between Mn intake and T2D risk

| **Moderator** | **logrr (95% CI)** | **SE** | **p-value** |
| --- | --- | --- | --- |
| Intercept | -0.37 (-1.38, 0.64) | 0.51 | 0.47 |
| Dietary assessment tool (FFQ) | 0.15 (-0.40, 0.70) | 0.28 | 0.59 |
| Study region (N. America) | -0.20 (-0.75, 0.35) | 0.28 | 0.48 |
| Sex (Female) | 0.03 (-0.04, 0.10) | 0.03 | 0.37 |
| Sex (Male) | 0.04 (-0.03, 0.12) | 0.04 | 0.26 |
| Follow-up (years) | 0.03 (-0.06, 0.12) | 0.05 | 0.54 |

Abbreviations: CI, confidence interval; FFQ, food frequency questionnaire; logRR, log relative ratio; SE, standard error

# Supplementary Fig. S1 Participant selection Flowchart for UKB analysis

Overall UKB population (N=502,370)

UKB participants without missing covariates (N=172,868)

With T2D at baseline (N=6,971)

T2D diagnosed before dietary assessment (N=703)

T2D analytical cohort (N=165,194)

CVD analytical cohort (N=164,111)

With CVD at baseline (N=7,104)

CVD diagnosed before dietary assessment (N=1,653)

Without Townsend deprivation index at recruitment (N=265)

Without BMI (N=3,267)

Without smoking (N=84)

Without drinking (N=85)

Without IPAQ (N=32211)

Without education (N=84)

Without baseline medication (N=94)

Without energy intake (N=19)

Implausible energy intake (N=784)

Without family history (N=2,734)

UKB participants with at least one dietary record (N=210,950)

Participants without dietary records (N=291,420)

# Supplementary Fig. S2 Flow chart of study selection process for systematic review and meta-analysis

Records removed *before screening*:

Duplicate records removed (n = 766)

Records identified from PubMed, Web of Science, Embase, Scopus (n = 2566)

**Identification**

Records excluded:

irrelevant topics (n =1537)

irrelevant exposures (n = 118)

review papers (n = 58)

Non-cohort observational studies (n = 39)

interventional studies (n = 31)

irrelevant outcomes (n = 5)

protocols (n = 5)

*conference abstracts (n = 2)*

*editorial (n = 1)*

Records screened

(n = 1800)

**Screening**

Reports sought for retrieval

(n = 4)

Reports not retrieved

(n = 0)

Reports excluded (n = 0)

Reports assessed for eligibility

(n = 4)

Added from reference list of included study (n = 1)

Studies included in review

(n = 5)

**Included**

# Supplementary Fig. S3 Restricted cubic spline for dose-response meta-analysis of original dietary manganese intake and hazard ratio of T2D incidence for overall sample, men, and women.


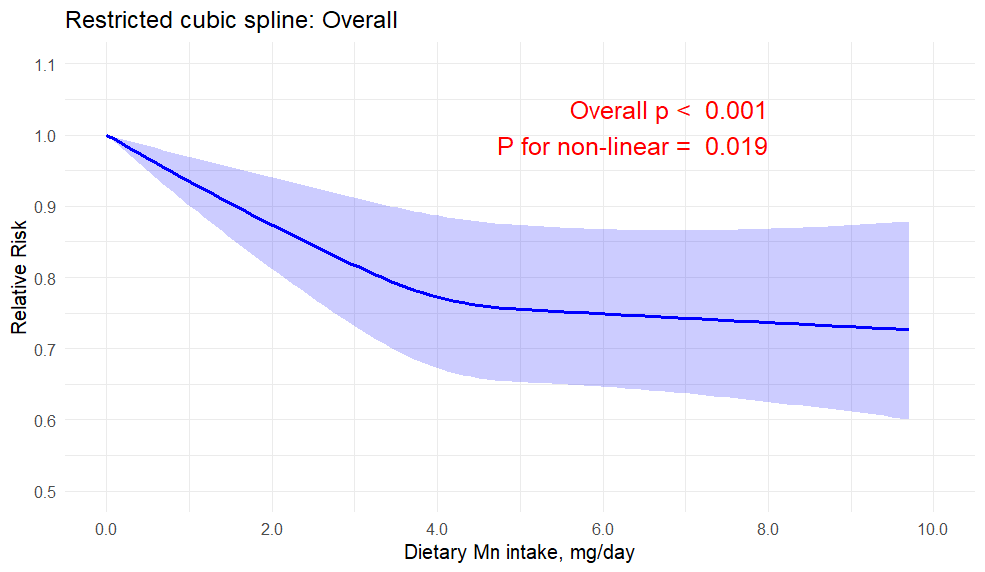

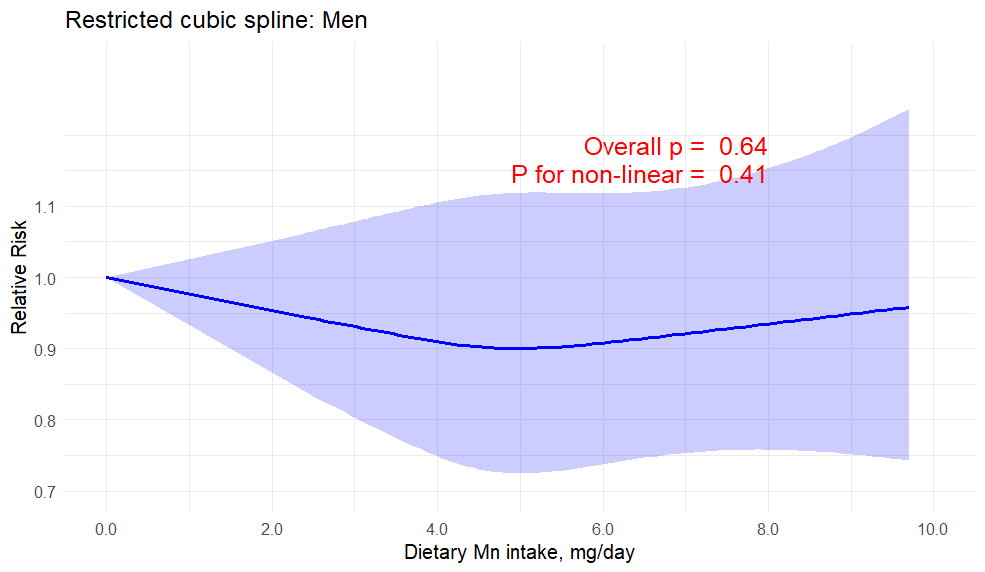

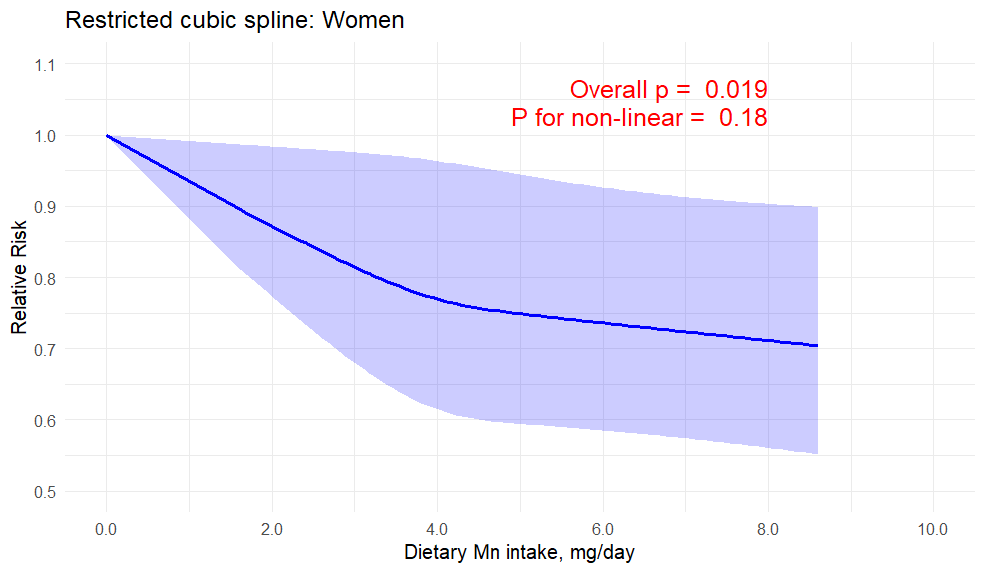


# Supplementary Fig. S4 Leave-one-out sensitivity analysis plot for the dose-response meta-analysis on dietary manganese and T2D risk.


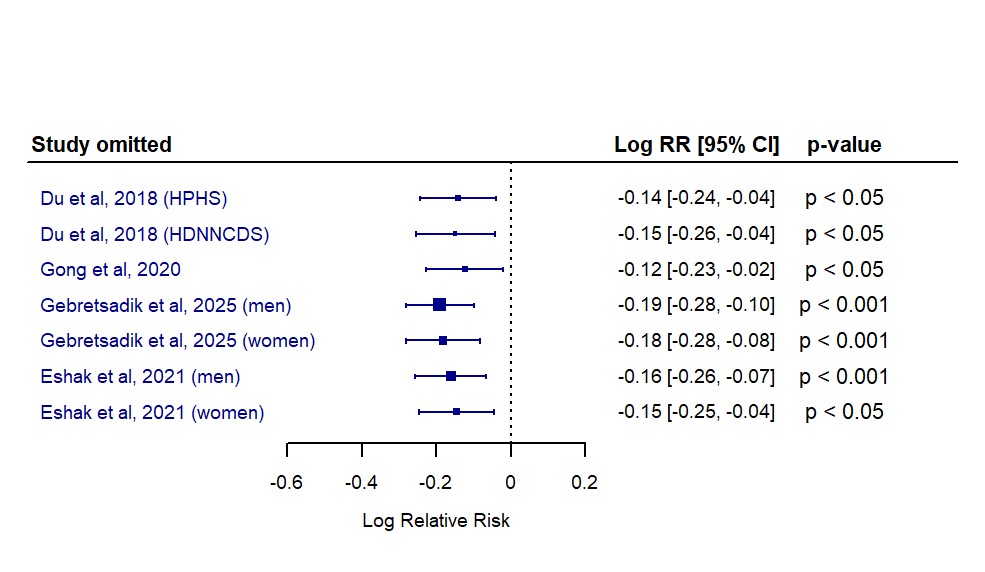


Each point represents the pooled effect estimate (logrr) after excluding one study, with 95% CI.

#

# Supplementary Fig. S5 Funnel plot with Egger’s regression line for publication bias in T2D meta-analysis

**
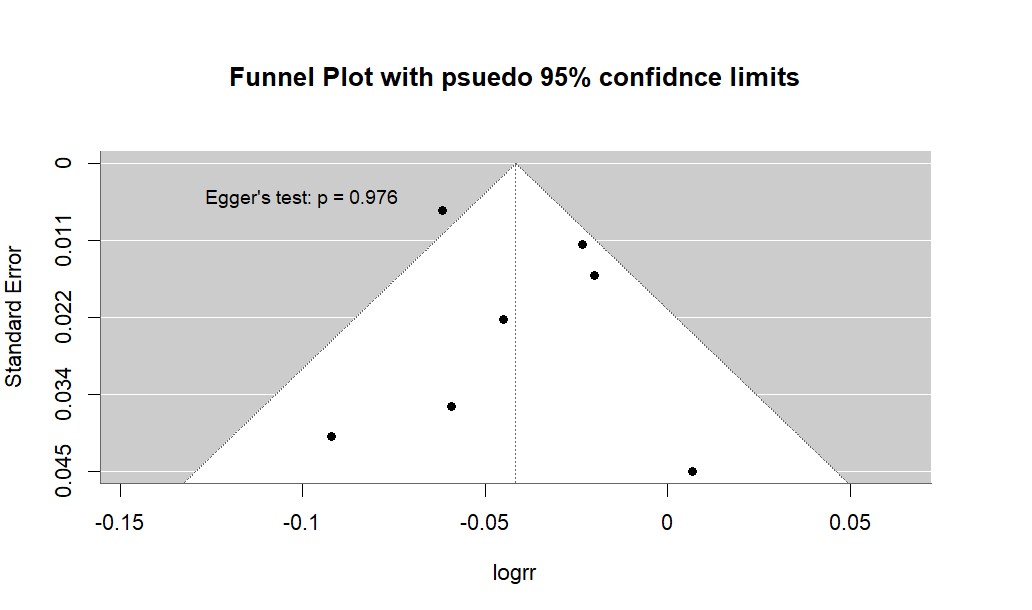
**

# Supplementary Fig. S6 Restricted cubic spline for dose-response meta-analysis of original dietary manganese intake and hazard ratio of total CVD for overall sample (a), men (b), and women (c).

1. Overall
2. Men
3. Women


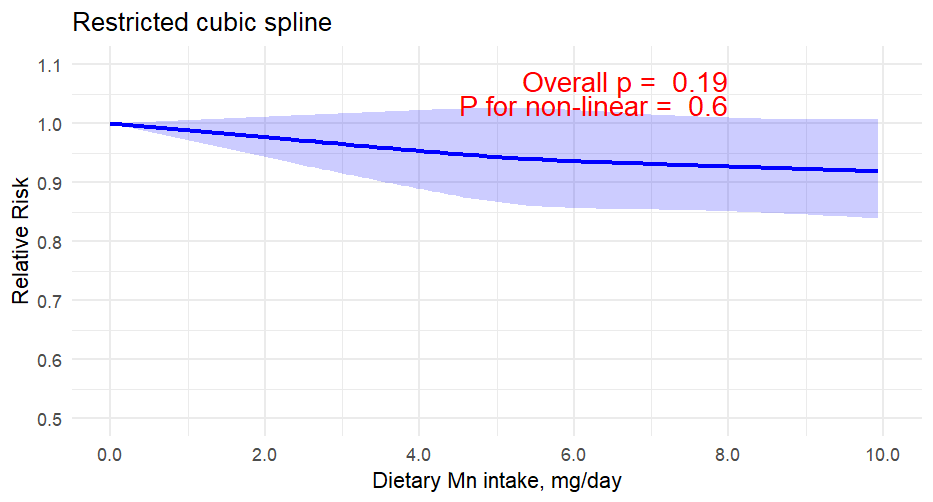

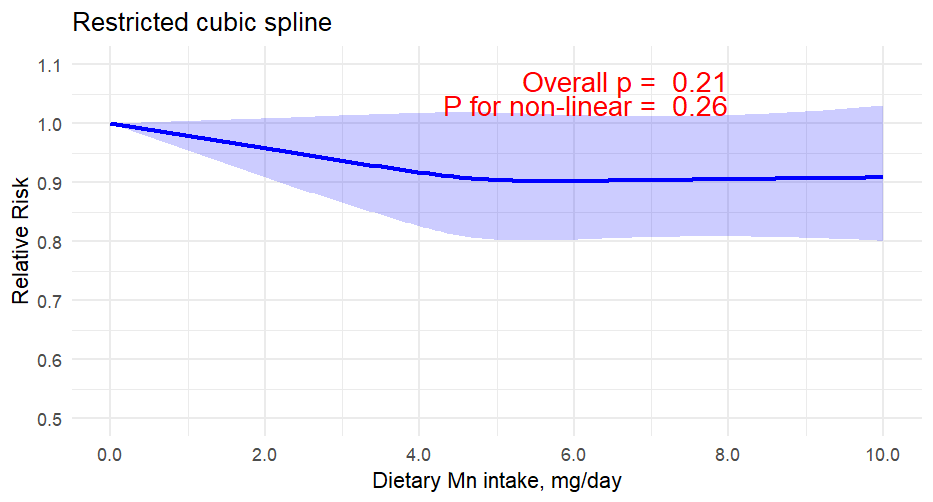

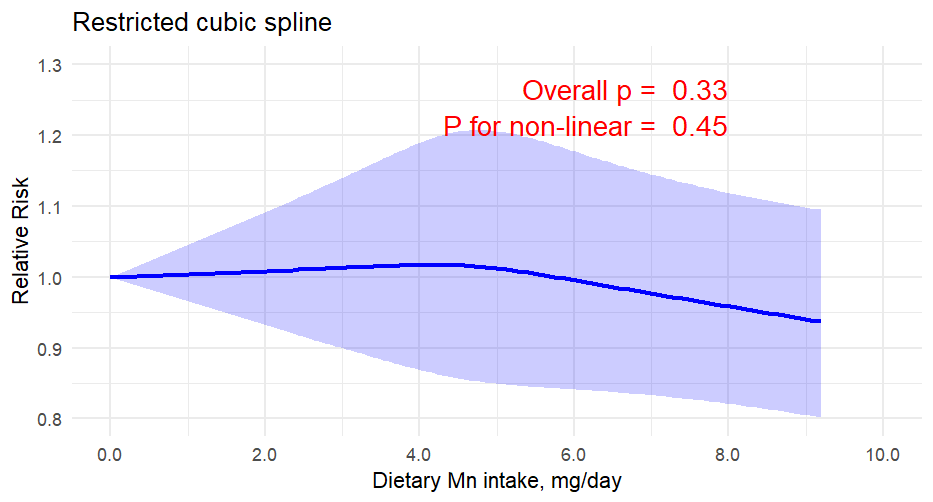


# Supplementary References

[1] Greenwood DC, Hardie LJ, Frost GS, Alwan NA, Bradbury KE, Carter M, et al. Validation of the Oxford WebQ online 24-hour dietary questionnaire using biomarkers. American journal of epidemiology. 2019;188(10):1858-67.

[2] Carter JL, Lewington S, Piernas C, Bradbury K, Key TJ, Jebb SA, et al. Reproducibility of dietary intakes of macronutrients, specific food groups, and dietary patterns in 211 050 adults in the UK Biobank study. Journal of Nutritional Science. 2019;8:e34.

[3] Perez-Cornago A, Pollard Z, Young H, van Uden M, Andrews C, Piernas C, et al. Description of the updated nutrition calculation of the Oxford WebQ questionnaire and comparison with the previous version among 207,144 participants in UK Biobank. European journal of nutrition. 2021;60(7):4019-30.

[4] Cleland C, Ferguson S, Ellis G, Hunter RF. Validity of the International Physical Activity Questionnaire (IPAQ) for assessing moderate-to-vigorous physical activity and sedentary behaviour of older adults in the United Kingdom. BMC medical research methodology. 2018;18:1-12.

[5] Chiuve SE, Fung TT, Rimm EB, Hu FB, McCullough ML, Wang M, et al. Alternative dietary indices both strongly predict risk of chronic disease. The Journal of nutrition. 2012;142(6):1009-18.

[6] Wright ME, Mayne ST, Stolzenberg-Solomon RZ, Li Z, Pietinen P, Taylor PR, et al. Development of a comprehensive dietary antioxidant index and application to lung cancer risk in a cohort of male smokers. American journal of epidemiology. 2004;160(1):68-76.

[7] Kang B, Yin X, Chen D, Wang Y, Lv J, Zhou J, et al. Balanced diets are associated with a lower risk of type 2 diabetes than plant-based diets. Diabetes Research and Clinical Practice. 2025;219:111977.

[8] Kivimäki M, Batty GD, Singh-Manoux A, Britton A, Brunner EJ, Shipley MJ. Validity of Cardiovascular Disease Event Ascertainment Using Linkage to UK Hospital Records. Epidemiology. 2017;28(5):735-9.

[9] Desquilbet L, Mariotti F. Dose-response analyses using restricted cubic spline functions in public health research. Stat Med. 2010;29(9):1037-57.
